# Metabolomics Analysis of Laparoscopic Surgery Combined with Wuda Granule to Promote Rapid Recovery of Patients with Colorectal Cancer Using UPLC/Q-TOF-MS/MS

**DOI:** 10.1155/2020/5068268

**Published:** 2020-02-13

**Authors:** Tao Wang, Yihua Xu, Qicheng Chen, Weilin Zheng, Jie Wang, Haiping Zeng, Yuyan Wu, Lixing Cao, Zhiqiang Chen

**Affiliations:** ^1^The Second Clinical College, Guangzhou University of Traditional Chinese Medicine, Guangzhou 510006, China; ^2^The Key Laboratory of Molecular Biology, State Administration of Traditional Chinese Medicine, School of Traditional Chinese Medicine, Southern Medical University, Guangzhou 510515, China; ^3^The Second Affiliated Hospital of Guangzhou University of Traditional Chinese Medicine, Guangzhou 510120, China

## Abstract

Surgery is the primary curative treatment for patients with nonmetastasized colorectal cancer (CRC). Rate of complications, morbidity, mortality, and overall survival of patients with CRC are factors associated with speed of recovery following surgery. Wuda granule (WD) is a traditional Chinese medicine (TCM) prescription used to promote rapid recovery after surgery. However, the specific mechanism of action of WD has not been characterized. Our study included 60 patients with clear histopathological evidence of colon or rectal cancer who underwent CRC laparoscopic surgery and 30 healthy individuals. Serum biochemistry and clinical evaluation of gastrointestinal function showed that WD could improve the nutritional status and gastrointestinal function and reduce the level of inflammation of patients with CRC following laparoscopic surgery. In addition, we used UPLC/Q-TOF-MS/MS-based metabolomics analysis to determine the mechanism of WD-related rapid recovery following laparoscopic surgery in patients with CRC. Twenty metabolites associated with arachidonic acid, alanine, aspartate and glutamate, *α*-linolenic acid, pyruvate, histidine, and glycerophospholipids were identified. The results suggested that the therapeutic mechanism of laparoscopic surgery combined with WD may be related to regulation of nutritional status, inflammation, immune function, energy, and gastrointestinal function in patients with CRC. This study also highlighted the ability of TCM compounds to interact with multiple targets to induce synergistic effects. This study may result in further studies of WD as a therapeutic agent to promote recovery following surgical resection of CRC tumors.

## 1. Introduction

Colorectal cancer (CRC) is an aggressive malignancy and can seriously affect the human colon and the rectum [1, 2]. Colorectal cancer causes 700,000 human deaths each year and has the fourth highest mortality rate, after lung cancer, liver cancer, and stomach cancer [3]. Incidence of CRC is low in individuals younger than 50 years of age, but the risk of developing CRC increases gradually with age. However, recent studies have indicated that the incidence of CRC in the younger could be increasing [4]. Historically, the incidence of CRC in East Asia and Eastern Europe was lower than that in the USA and Western developed countries. However, improved living standards and a more westernized lifestyle have resulted in a rapid increase in the incidence of CRC in East Asia and Eastern Europe [5]. In recent years, the incidence of CRC has continued to rise in China, particularly in rural areas where medical resources are lacking [6]. Clinical treatment strategies for CRC include surgery, chemotherapy, and radiotherapy. Surgery is the primary curative treatment for patients with nonmetastasized CRC [7]. Development of minimally invasive surgical techniques and advancement in laparoscopic equipment have allowed for widespread use of laparoscopic radical resection of CRC. Compared with traditional open surgery, laparoscopic CRC surgery results in fewer complications, faster recovery, lower recurrence rates, and longer survival. In addition, the internationally recognized enhanced recovery after surgery (ERAS) protocol has been shown to reduce the length of hospital stays and risk of complications related to colorectal surgery [8, 9]. However, rehabilitation following laparoscopic CRC surgery is still a major obstacle, and complications such as postoperative ileus (POI) can occur. Delayed recovery of gastrointestinal function directly affects the overall recovery of patients with CRC following surgery, and delayed return of function is associated with complications, morbidity, mortality, and overall survival of patients with CRC. In addition, delayed recovery of gastrointestinal function can prolong hospitalization time, increase hospitalization costs, reduce the utilization rate of medical resources, and can be a significant social and economic burden.

No effective drugs are available to promote rapid rehabilitation following surgery. Recovery of gastrointestinal motility is an important predictor of recovery rate following surgery. Commonly used gastrointestinal motility drugs such as Motilium, cisapride, and metoclopramide can promote recovery of gastrointestinal function, but use of these drugs is limited by significant risk of adverse cardiovascular and extrapyramidal reactions [10–12]. Recently, alvimopan, a *μ*-type opioid receptor antagonist, was approved for use in the United States. Alvimopan can improve colonic motility, but its efficacy is limited, and it is not widely used [13–15]. Current postoperative management relies mainly on indirect measures such as reducing surgical trauma and stimulation and postoperative gastrointestinal decompression. Current active intervention methods are inadequate.

Recently, traditional Chinese medicine (TCM) has become more widely used in clinical practice. Traditional Chinese medicines are beneficial for regulation of immune function, enhancing curative effects, reducing adverse reactions, and eliminating drug resistance in patients with cancer. Wuda granule (WD) (formerly Xiangbin prescription) is currently used as an in-hospital preparation at the Guangdong Provincial Hospital of TCM (the Second Affiliated Hospital of Guangzhou University of TCM). Our previous studies showed that WD may promote rapid recovery following surgical procedures [16–18]. Wuda granule is a TCM prescription comprised of *Panax ginseng* (Renshen), *Fructus amomi* (Sharen), *Areca catechu* L. (Binlang), *Lindera aggregata* Kosterm (Wuyao), and *Prunus persica* Batsch (Taoren). Previous studies used liquid chromatography multiple-reaction-monitoring mass spectrometry (LC-MRM-MS) to simultaneously determine the content of the 10 components (ginsenoside Rc, ginsenoside Rd, ginsenoside Rg1, quercetin, quercitrin, isoquercitrin, laetrile, norisoboldine, linderane, and arecoline) in WD [19], and ultrahigh performance liquid chromatography coupled to triple quadrupole mass spectrometry (UPLC-QqQ-MS/MS) was used to simultaneously identify four alkaloids in WD [20]. Characterization of the composition of WD provided a scientific basis for quality control of this medicinal compound. However, the specific mechanisms of WD have not been characterized.

Metabonomics is a discipline developed in the 1990s and is patterned after genomics, transcriptomics, and proteomics. In addition, metabonomics is an important technique in systems biology [21], in that it is used to study the organism as a whole, which is consistent with the holistic view of TCM and the concept of syndrome differentiation and treatment [22]. Metabonomics accounts for the influences of internal and external factors on biological metabolic networks and is an effective research method to evaluate the overall efficacy of TCM compounds. Ultraperformance liquid chromatography with electrospray ionization quadrupole time-of-flight tandem mass spectrometry (UPLC/Q-TOF-MS/MS) allows for highly specific and sensitive identification and quantitation of hundreds of molecular species within a single biological sample. UPLC/Q-TOF-MS/MS-based metabolomics is particularly suitable for metabolite identification [23]. Therefore, we used a UPLC/Q-TOF-MS/MS-based metabolomics approach to characterize the mechanism of WD-induced rapid recovery in patients with CRC following laparoscopic surgery.

## 2. Materials and Methods

### 2.1. Ethical Statement

Informed consent was obtained from all participants, and the study protocol was approved by the Ethics Committee of Guangdong Provincial Hospital of TCM. The approval number for ethical review was YF2018-192-01. The privacy of all human subjects was protected throughout the study.

### 2.2. Preparation of WD

Wuda granule is currently an in-hospital preparation at Guangdong Provincial Hospital of TCM and has been produced and marketed within this hospital. Wuda granule is comprised of *Fructus amomi* (Sharen) 300 g, *Lindera aggregata* Kosterm (Wuyao) 500 g, *Panax ginseng* (Renshen) 450 g, *Prunus persica* Batsch (Taoren) 500 g, and *Areca catechu* L. (Binlang) 500 g. It is produced in 1000 g batches and distributed in 10 g/bag units. One bag of WD is administered orally, twice daily, starting 6 h after surgery.

### 2.3. Instruments and Reagents

An AB Sciex 5600 Triple-TOFTM mass spectrometer with Analyst®TF 1.7 software, PeakView 2.0 data processing software, and Markerview metabonomics analysis software (AB Sciex, CA, USA) were used for data collection. SIMCA 14.0 multivariate statistical software (Umetrics, Sweden) was used for data analysis. The LC-30AD ultrahigh performance liquid chromatography system was comprised of a SIL-30AC autosampler, a DGU-20ASR online degasser, a CTO-20AC temperature-controlled column manager, and a CBM-20A pump (all from Shimadzu, Kyoto, Japan). An Acquity UPLC HSS T3 column (Waters, USA; 100 × 2.1 mm, 1.7 *μ*m) was used for separation. Acetonitrile, methanol (Merck, Germany), and formic acid (Sigma-Aldrich, USA) were of LC-MS grade. Water used in this study was ultrapure water (Millipore, MA, USA).

### 2.4. Study Subjects and Serum Sample Collection

Sixty patients with clear histopathological evidence of colon or rectal cancer who underwent colorectal cancer surgery from March 2015 to June 2018 in Guangdong Provincial Hospital of TCM (The Second Affiliated Hospital of Guangzhou University of TCM) were included in this study. The duration of the surgical procedure was 1–4 h for all patients, and the time under anesthesia was 1.5–4.5 h. No patients experienced serious complications, infections, adverse events, or secondary surgery during treatment. All patients received the same fundamental treatment after surgery, including antibiotic therapy, rehydration support therapy, and exposure to a rapid rehabilitation program. Thirty patients in the WD treatment group received WD orally after surgery until they defecated. Thirty healthy individuals who came to Guangdong Provincial Hospital of TCM for physical checkups were included as controls. Serum samples from the control patients were tested, and all routine indicators were within normal ranges, and no other diseases were present.  Table 1 summarizes the characteristics of the samples. Subjects were divided into four groups: healthy controls (HG), patients with colorectal cancer (model group, MG), routine fundamental treatment following laparoscopic surgery group (FTG), and the WD treatment group (WDG). HG has 30 healthy individuals, MG has 60 patients of CRC, and FTG and WDG have 30 postoperative patients, respectively. Blood samples were collected from patients who underwent CRC surgery before the procedure and on the morning of the third day after surgery. After 10 min of centrifugation at 4°C (3,000 rpm), 250 *μ*L of supernatant was transferred to a fresh centrifugal tube and stored at –80°C until use.

### 2.5. Sample Preparation

One hundred microliters of serum and 300 *μ*L of precooled methanol solution were transferred to a 1.5 mL centrifuge tube. After 5 min of vortex mixing, the samples were centrifuged at 14,000 rpm for 10 min. The supernatant was transferred to a vial for nontargeted metabonomics analysis. In addition, 30 *μ*L of each sample was placed in a vial for use as a quality control sample.

### 2.6. Metabonomics Analysis

The UPLC instrument and the column are described in Section 2.3. Mobile phase A was acetonitrile with 0.1% formic acid, and mobile phase B was water with 0.1% formic acid. The gradient elution conditions were as follows: 0.0–3.0 min, 5–20% A; 3.0–7.0 min, 20–60% A; 7.0–10.5 min, 60–75% A; 10.5–14.5 min, 75–95% A; 14.5–15.0 min, 95–100% A; 15.0–18.0 min, 100% A; 18.0–18.5 min, 100–5% A; and 18.5–21.5 min, 5% A. The flow rate was 0.4 mL/min, the column was maintained at 40°C, and the injection volume was 5 *μ*L.

An AB Sciex 5600 Triple-TOF high-resolution mass spectrometer equipped with an electrospray ion source was used to collect data in the positive and negative mode. The mass spectrometer operating conditions were as follows: the atomizing gas and the auxiliary heating gas were set to 50 psi, the air curtain gas was 30 psi, the spray voltage was 4500 V (positive mode) or –4500 V (negative mode), the declustering potential was 90 V (positive mode) or –100 V (negative mode), the source temperature was 500°C, the collision energy was 30 eV (positive mode) or –30 eV (negative mode), and the range of collision energy expansion was 15 eV. The scan range was 40–1000 *m*/*z*. All other parameters were operated under default conditions. The information-dependent acquisition (IDA) was used for data collection. Under these conditions, 10 secondary mass spectrometry data were scanned simultaneously with the first-level mass spectrometry data, and background subtraction was performed. During operation, the calibration delivery system (CDS) was corrected automatically at every 3 h to ensure the quality and accuracy of the data.

### 2.7. Metabonomics Data Processing and Statistical Analysis

The integrity and quality of the collected data were evaluated using PeakView software. Then, the data from each group of samples were imported into Markerview data processing software for peak alignment and area normalization. Two-dimensional data consisting of retention time, mass number, and mass spectrum intensity were compiled. The parameters used in Markerview data processing software were as follows: the minimum chromatographic peak width was 6 scans, the minimum mass spectrum peak width was 35 ppm, the retention time deviation was less than 12 s, the signal-to-noise ratio was greater than 100, the mass deviation was less than 5 ppm, and the number of samples in which the peak appeared was greater than 30. The aligned and normalized data were imported into SIMCA 14.0 data processing software for multivariate statistical analysis, which included PCA (unsupervised principal component analysis) and OPLS-DA (supervised orthogonal partial least squares discriminant analysis). Principal component analysis was used to identify any abnormal values and to determine any differences between the MG and the HG; the FTG and the MG; the WDG and the MG; the FTG and the HG; the WDG and the HG; and the WDG and the FTG. OPLS-DA analysis was performed between the two groups to evaluate the differences between the MG and the HG, the FTG and the MG, and the WDG and the MG and to generate corresponding S-plots and VIP graphs. The points at each end of the S-plot diagrams are potential differentially abundant metabolites. Metabolites with VIP > 1.5 and one-way ANOVA values with *p* < 0.05 were used to identify differences in metabolite levels between groups. The chemical structures of metabolites with differential abundance between groups were identified through comparison with the literature, the HMDB database (http://www.hmdb.ca/), the KEGG database (https://www.kegg.jp/), and the METLIN database (https://metlin.scripps.edu/). Finally, differentially abundant metabolites were imported into MetaboAnalyst 4.0 online analysis software (https://www.metaboanalyst.ca/) for pathway enrichment, and KEGG data were used to determine the statistical significance of related pathways and possible mechanisms of WD-related rapid recovery following laparoscopic surgery in patients with CRC.

## 3. Results

### 3.1. Analysis of Biochemical Indicators

**Figure 1:**
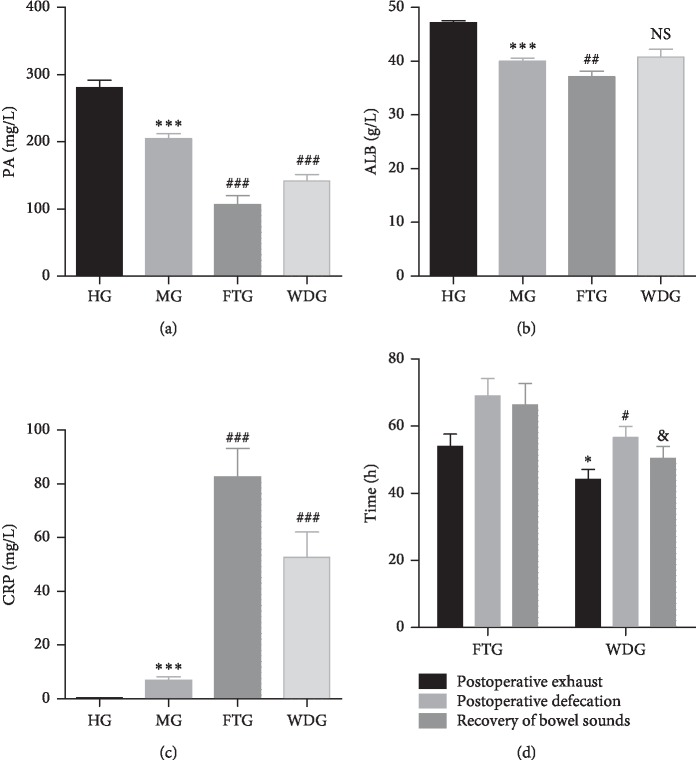
PA, ALB, and CRP levels in HG, MG, FTG, and WDG, and the recovery of gastrointestinal function in FTG and WDG. (a) PA levels in HG, MG, FTG, and WDG. (b) ALB levels in HG, MG, FTG, and WDG. (c) CRP levels in HG, MG, FTG, and WDG (^*∗∗∗*^*p* < 0.001, MG vs. HG; ^##^*p* < 0.01, ^###^*p* < 0.001, FTG, WDG vs. MG). (d) Postoperative exhaust (^*∗*^*p* < 0.05, WDG vs. FTG), postoperative defecation (^#^*p* < 0.05, WDG vs. FTG), and recovery of bowel sound time (^&^*p* < 0.05, WDG vs. FTG) for FTG and WDG.

Serum levels of prealbumin (PA), albumin (ALB), and C-reactive protein (CRP) in the HG, MG, FTG, and WDG groups were quantified. Detailed results are summarized in Figures 1(a)–1(c). Prealbumin and ALB levels were significantly lower in the MG group than those in the HG group, and CRP was significantly increased in the MG group compared with the HG group. Prealbumin levels were significantly lower in the FTG and WDG groups than those in the MG group, and ALB levels were significantly lower in the FTG group than those in the MG group. There was no significant difference in ALB level between the WDG and MG groups. In contrast, CRP levels were significantly higher in the FTG and WDG groups than those in the MG group. It can be found that WDG tends to approach HG more than FTG under the same downward or upward trend of FTG and WDG. These results indicated that laparoscopic surgery combined with WD resulted in the greatest therapeutic effect.

### 3.2. Effect of WD on Recovery of Gastrointestinal Function in Patients with CRC following Laparoscopic Surgery

Postoperative recovery of gastrointestinal function was evaluated using the time of first exhaust, the time of first defecation, and the time to recovery of bowel sounds as indicators of recovery. Because the HG and MG groups did not undergo operations, only the FTG and WDG groups were compared. The results for time to postoperative exhaust, postoperative defecation, and recovery of bowel sounds in the FTG and WDG groups are summarized in Figure 1(d). The time to each of these three indicators was significantly shorter in the WDG group than that in the FTG group (*p* < 0.05), which indicated that WD treatment promoted faster recovery of gastrointestinal function.

### 3.3. Serum Metabolite and Quality Control Analyses

**Figure 2:**
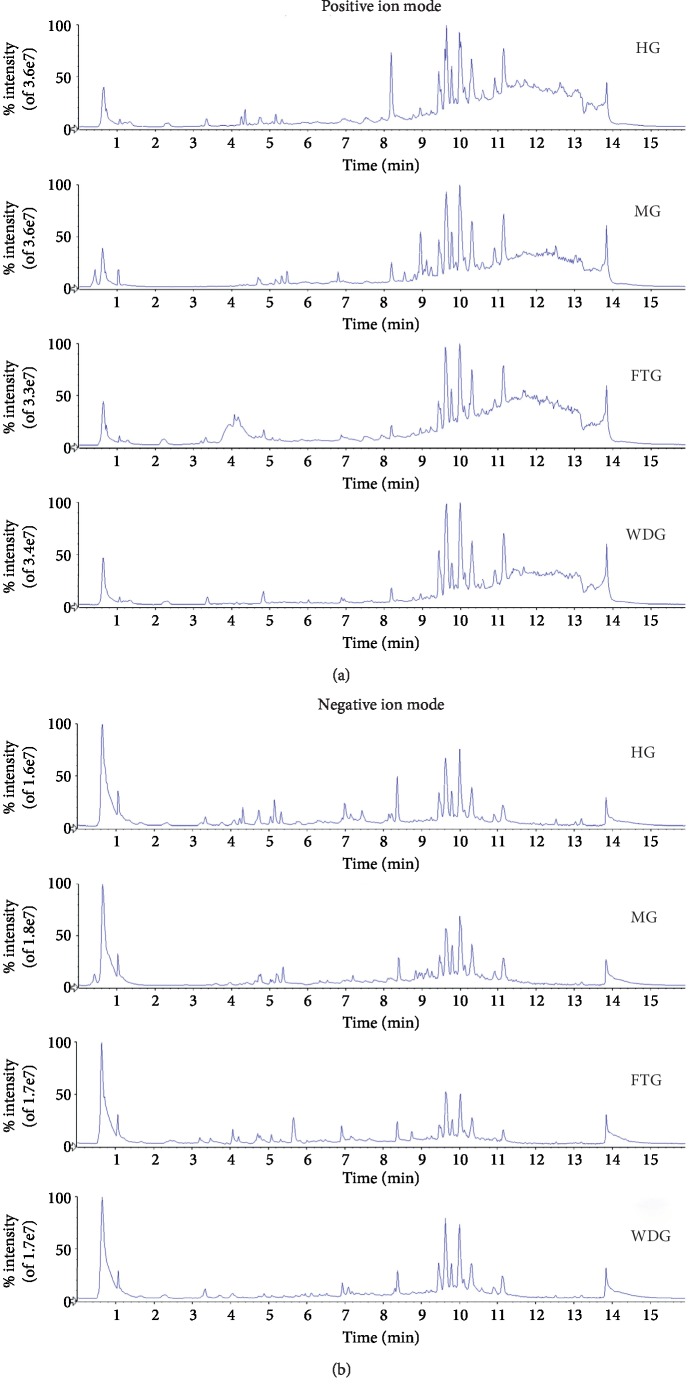
Representative total ion chromatogram (TIC) of HG, MG, FTG, and WDG in positive and negative ion modes. (a) Positive ion mode. (b) Negative ion mode.

Serum samples from subjects in the HG, MG, FTG, and WDG groups were analyzed using UPLC-Q-TOF-MS/MS. Representative total ion chromatograms (TIC) for each group in positive and negative modes are shown in Figure 2. The data were imported into PeakView software, which showed that the numbers and intensities of ions were different among the groups. The metabolic profiles between the MG and HG groups were significantly different, and the metabolic profiles between the FTG and WDG groups were also significantly different. In addition, 30 *μ*L of each sample was placed in a vial for use as a quality control (QC) sample. During mass spectrometry data acquisition, four QC samples were repeatedly injected before injection of samples, and one QC sample was injected after every five samples. Total ion chromatograms of the QC samples in the positive and the negative ion mode are shown in [Supplementary-material supplementary-material-1]. The results showed that the QC samples had good repeatability. In addition, six representative metabolites were selected to evaluate repeatability and stability in the positive and the negative mode. The percent relative standard deviations (%RSD) of the retention times and the intensities of the six representative metabolites in the positive and the negative mode were less than 1.1% and 11.0%, respectively (Table 2). These results indicated that the analytical method had good repeatability and was stable across the entirety of the injection sequence.

### 3.4. Multivariate Statistical Analysis

**Figure 3:**
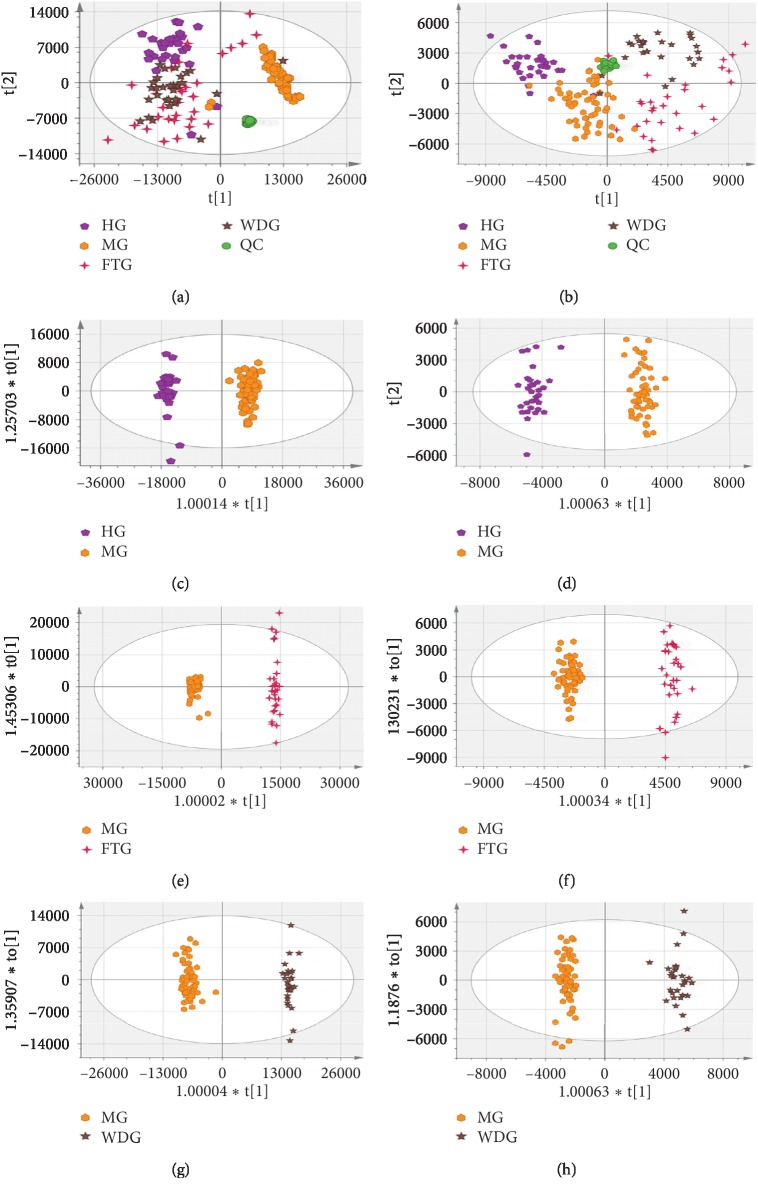
PCA and OPLS-DA score plots in the positive mode and the negative mode. PCA score plot of each group in the positive mode (a) and the negative mode (b). OPLS-DA score plots from MG vs HG in the positive mode (c) and the negative mode (d); FTG vs MG in the positive mode (e) and the negative mode (f); and WDG vs MG in the positive mode (g) and the negative mode (h). The model parameters were (a) R2X = 0.836, Q2 = 0.606; (b) R2X = 0.884, Q2 = 0.671; (c) R2X = 0.615, R2Y = 0.983, Q2 = 0.968; (d) R2X = 0.754, R2Y = 0.942, Q2 = 0.840; (e) R2X = 0.677, R2Y = 0.992, Q2 = 0.963; (f) R2X = 0.664, R2Y = 0.930, Q2 = 0.788; (g) R2X = 0.691, R2Y = 0.988, Q2 = 0.958; and (h) R2X = 0.725, R2Y = 0.914, Q2 = 0.870.

**Figure 4:**
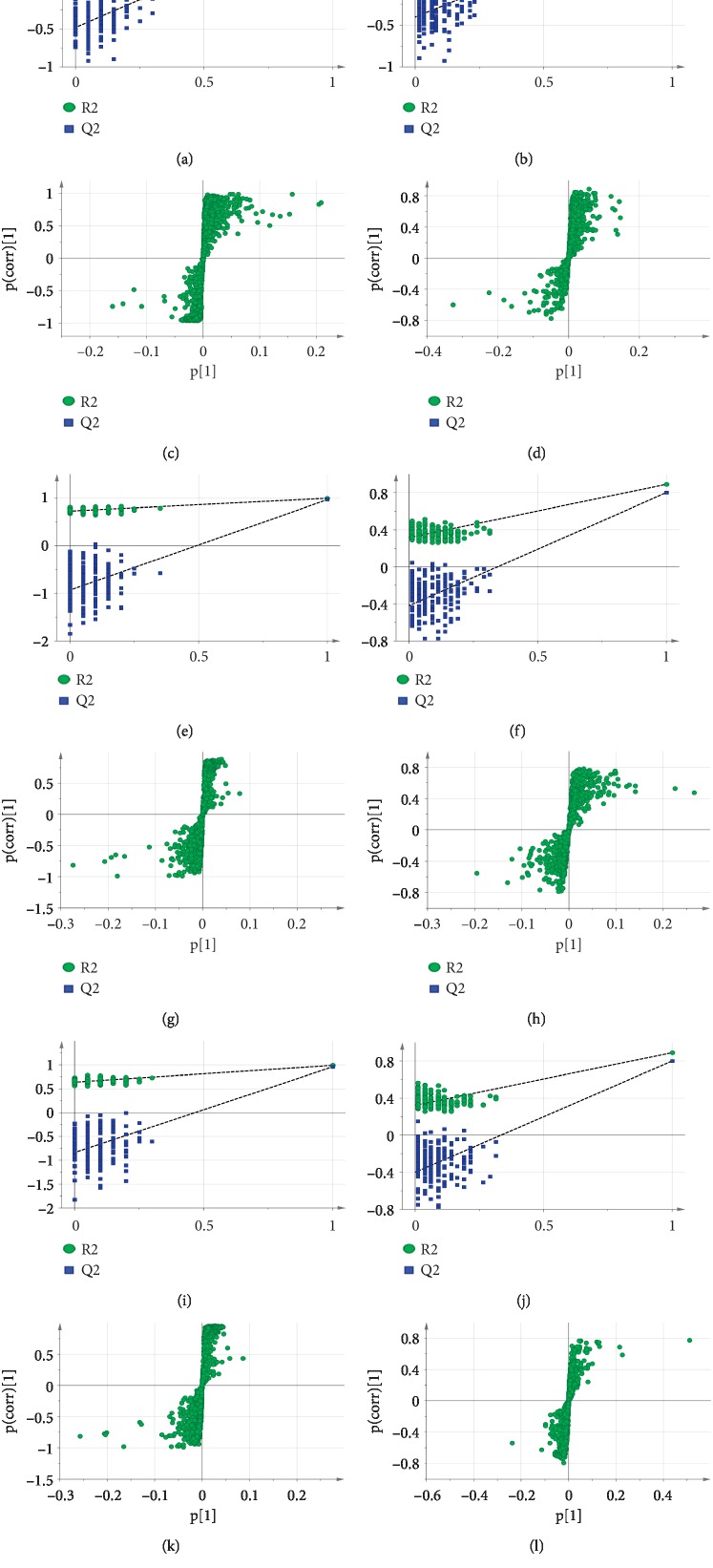
Validation of the OPLS-DA model by permutation tests with 200 times and generated S-plot. 200-time permutations were performed and plotted and compared MG vs HG in (a) positive ion and (b) negative ion, FTG vs MG in (e) positive ion and (f) negative ion, and WDG vs MG in (i) positive ion and (j) negative ion. The yielded intercept values of (a) R2 = 0.358, Q2 = –0.481; (b) R2 = 0.493, Q2 = –0.536; (e) R2 = 0.726, Q2 = –0.932; (f) *R*2 = 0.478, Q2 = –0.593; (i) R2 = 0.639, Q2 = –0.838; and (j) R2 = 0.314, Q2 = –0.393. OPLS-DA S-plot for serum metabolites compared MG vs HG in (c) positive ion and (d) negative ion, FTG vs MG in (g) positive ion and (h) negative ion, and WDG vs MG in (k) positive ion and (l) negative ion.

Following noise reduction, peak alignment, and normalization, the mass spectrometry data were imported into SIMCA 14.0 software for multivariate statistical analysis to evaluate changes in metabolic profiles between the MG and HG groups and between the WDG and FTG groups. Principal component analysis and OPLS-DA are frequently used to determine differences between experimental groups and to screen differences that contribute to the differing characteristics of the groups. Data quality was evaluated first. Four groups of unsupervised PCA models of serum samples in the positive and the negative ion mode were established, as shown in Figures 3(a) and 3(b). The results showed that there were clear differences between the MG and HG groups, the FTG and MG groups, and the WDG and MG groups. Furthermore, the QC samples clustered tightly in the PCA score plot, which indicated that the system had stability across the injection sequence. Supervised OPLS-DA analysis was then performed to further characterize differences between groups and to determine which variables explained the differences between the groups. The results showed that the HG, FTG, and WDG groups were significantly different than the MG group in both positive and negative modes (Figures 3(c)–3(h)). The R2 and Q2 values suggested that the OPLS-DA model provided excellent fits and was highly predictive. The intercept values for each OPLS-DA model after 200 permutations indicated that the model was stable and reliable and did not suffer from overfitting (Figures 4(a) and 4(b), 4(e) and 4(f), and 4(i) and 4(j)).

### 3.5. Screening and Identification of Twenty Metabolites with Differential Abundances

Using the results of the OPLS-DA model, we generated S-plots for each model (Figures 4(c) and 4(d), 4(g) and 4(h), and 4(k) and 4(l)), in which differentially abundant metabolites were located at both ends of the S-shape. We then identified target metabolites as those with VIP > 1.5 and *p* < 0.05 in one-way ANOVA. The chemical structures of these metabolites were identified by comparison with the literature, HMDB, KEGG, and the Metlin database. Twenty metabolites were identified as potential biomarkers in the MG, FTG, and WDG groups (Table 3). The results showed that the levels of LysoPC (16:0), LysoPC (18:1), LysoPC (18:0), LysoPC (18:2), LysoPC (22:6), LysoPE (16:0), 11-HETE, leucine, cystine, LysoPE (22:6), arachidonic acid, pyruvic acid, palmitic acid, and 9-HODE were significantly increased (*p* < 0.01), and glutamine, 5-hydroxytryptamine, tryptophan, thromboxane B_2_, and *α*-linolenic acid were significantly decreased, in the MG group compared with those in the HG group (*p* < 0.01). Histidine levels were increased, but the difference was not statistically significant. The levels of LysoPC (16:0), LysoPC (18:1), LysoPC (18:0), LysoPC (18:2), LysoPC (22:6), LysoPE (16:0), 11-HETE, leucine, LysoPE (22:6), arachidonic acid, pyruvic acid, palmitic acid, and 9-HODE were significantly decreased (*p* < 0.05), and glutamine, tryptophan, and thromboxane B_2_ were significantly increased in the FTG and WDG groups compared with those in the MG group (*p* < 0.001). Furthermore, the levels of 5-hydroxytryptamine, linolenic acid, and histidine were significantly higher in the WDG group than those in the MG group (*p* < 0.05). Cystine significantly decreased only in the FTG group but not in the WDG group compared with that in the MG group (*p* < 0.05). This suggested that WD may induce metabolomic changes when administered in conjunction with laparoscopic surgery and basic postoperative treatment. The ability to regulate the level of twenty metabolites simultaneously to approach the HG that the WDG was more effective than the FTG, which may have contributed to the advantages of the multicomponent, multitarget, and multilevel actions of TCM.

**Figure 5:**
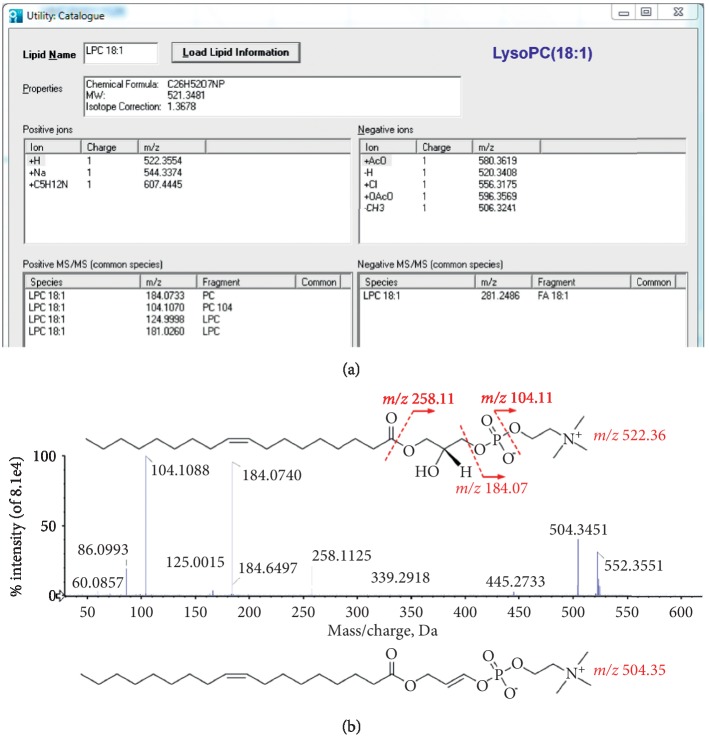
The fragmentation pathway of LysoPC (18:1) was well matched with the record in LipidView™ 1.2. (a) The record information of LysoPC (18:1) in LipidView™ 1.2. (b) Fragmentation pathway and MS/MS fragments of compound LysoPC (18:1) in the positive ion mode.

Identification of differentially abundant metabolites was performed primarily using online MS databases (Metlin, HMDB, and Lipid MAPS) and LipidView™ 1.2 software (AB Sciex, Foster City, CA). As an example, fragment ion *m*/*z* 522.3554 had a retention time of 10.29 min. The VIP value of the biomarker was 9.57. There were five peaks with high abundance in the product ion spectrum of *m*/*z* 522.3554 in the positive mode: *m*/*z* 522.3551, *m*/*z* 504.3451, *m*/*z* 258.1125, *m*/*z* 184.0740, and *m*/*z* 104.1088. We then searched the precursor and product masses of *m*/*z* 522.3554 in the HDMB database. The metabolite was identified as LysoPC (18:1), with the pseudomolecular ion formula C_26_H_52_NO_7_P[M+H]^+^ based on accurate mass (mass error < 5 ppm). We determined that the fragment ion *m*/*z* 504.3451 was likely formed by loss of neutral water from the pseudomolecular ion *m*/*z* 522.3554. The fragment ions *m*/*z* 258.1125 (glycerophosphocholine, chemical formula C_8_H_20_NO_6_P), *m*/*z* 184.0740 (phosphorylcholine, chemical formula C_5_H_15_NO_4_P), and *m*/*z* 104.1088 (choline, chemical formula C_5_H_14_NO) were formed through different losses from *m*/*z* 522.3554. The fragmentation pathway matched well with that in LipidView™1.2 (Figure 5(a)). The possible MS/MS fragmentation pathways are summarized in Figure 5(b). Thus, the ion with *m*/*z* 522.3554 was identified as LysoPC (18:1).

### 3.6. Metabolic Pathway Analysis

**Figure 6:**
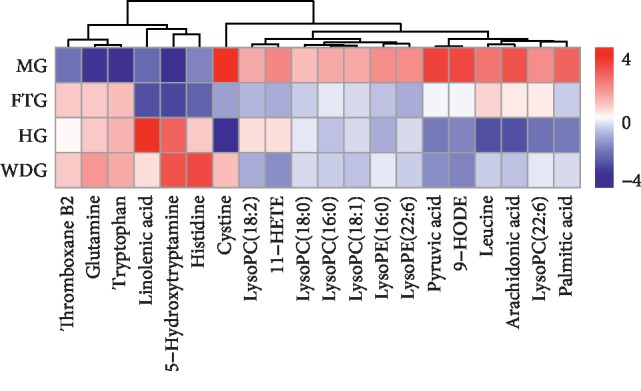
Heatmap analysis of the 20 identified metabolites between HG, MG, FTG, and WDG. The relative content of the compound is shown by color depth, the higher content is represented in red, and the lower content is represented in blue.

MetaboAnalyst 4.0 (https://www.metaboanalyst.ca/) is a web-based software that uses heatmap analysis to compare the relative content of metabolites. We compared the 20 metabolites identified in our study. The results showed that there were significant differences among the four groups, as shown in Figure 6. There were obvious differences between the MG and HG groups. In addition, the WDG group was more similar to the HG group than was the FTG group. These results showed that addition of WD treatment to laparoscopic surgery resulted in a metabolic profile much more similar to healthy controls than to patients that did not receive WD.

**Figure 7:**
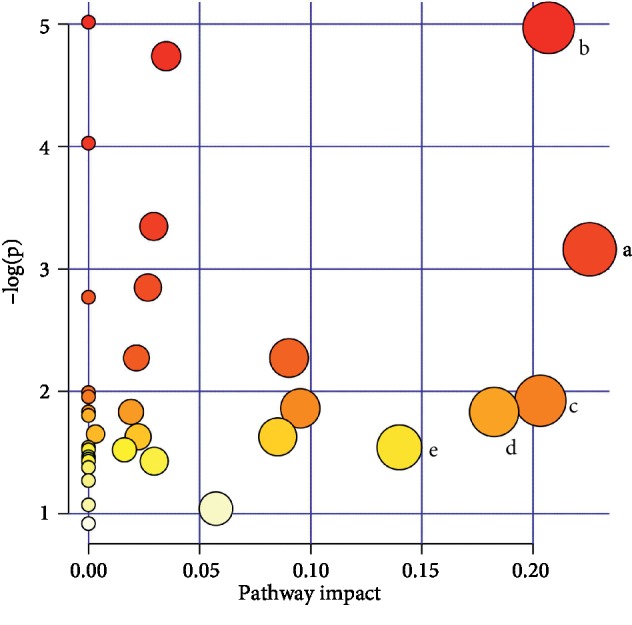
The result of metabolic pathway analysis in the four groups. The significantly relevant metabolic pathways: (a–e) the metabolic pathway details shown in Table 4.

**Figure 8:**
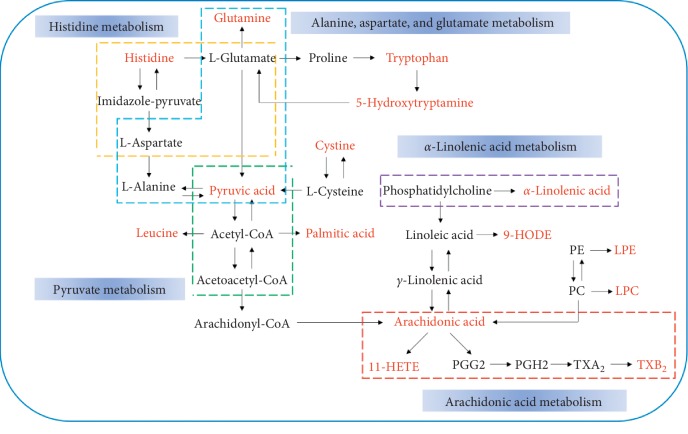
Metabolism pathway networks. Different dotted boxes represent different metabolic pathways. The name of the pathway is in the blue box next to the dotted line frame. Metabolite name with red bold represents the differential metabolites which were identified.

Next, differential metabolite pathway enrichment was performed using MetaboAnalyst software. The results of pathway enrichment are shown in Table 4 and Figure 7. There were five metabolic pathways with pathway impact values >0.1, which were considered significantly relevant: arachidonic acid metabolism; alanine, aspartate, and glutamate metabolism; alpha-linolenic acid metabolism; pyruvate metabolism; and histidine metabolism. Use of the KEGG database showed that the metabolic pathways were linked through the differentially abundant metabolites to form a metabolic pathway network, as shown in Figure 8. The compounds shown in red were identified as differentially abundant metabolites. Laparoscopic surgery combined with WD formed a complex network of metabolic pathways to promote rapid recovery in patients with CRC.

## 4. Discussion

Despite significant improvements in multimodal treatments over the last several decades, the removal of solid tumors continues to be the primary method of treating patients with cancer [24]. Therefore, rapid recovery of patients with cancer following surgery is an important issue. Postoperative rapid recovery is closely related to postoperative complications, morbidity, and mortality [25]. The rate at which patients with cancer can recover following surgery is closely related to nutritional status, levels of inflammation, immune function, energy, and gastrointestinal function.

Albumin is a polypeptide traditionally used as a quantitative measure of nutritional status because it is readily available and inexpensive. Furthermore, ALB has also been used as a biochemical marker of individual nutritional status prior to surgery [26]. Albumin levels are associated with complications and mortality in patients with cancer following surgery [27]. Prealbumin, also known as transthyretin, is a visceral protein and a negative acute-phase reactant similar to ALB. Prealbumin has a shorter half-life (2-3 days) than ALB, which makes PA a better indicator of acute changes in nutritional status. However, ALB is superior to PA for predicting short-term recurrence in patients with operable CRC [28]. Albumin and PA are among the most commonly used tools to measure nutritional status [29, 30]. C-reactive protein is an acute-phase protein with a half-life of 19 h. It is synthesized and secreted by the liver and is an effector of the inflammatory response. C-reactive protein and white cell count (WCC) are the most commonly used markers of postoperative inflammation and infection [31]. Patients with CRC who undergo surgery often suffer from malnutrition due to advanced malignant tumors, resulting in inadequate oral intake, intestinal obstruction, intestinal fistula, poor absorptive capacity, and massive loss of gastrointestinal tract [32]. Up to 80% of patients with advanced CRC suffer from malnutrition, which increases the risk of postoperative complications [33, 34]. Tumors depend on their host for sustenance and compete with the host for nutrients such as ALB, resulting in abnormal levels of ALB and PA, which can lead to malnutrition and weight loss. In addition, tumor growth might lead to visceral tissue inflammation and increased CRP levels [35]. Previous studies showed that there may be a significant association between inflammation and cancer [36]. Our results were consistent with these findings. The levels of ALB and PA in patients with CRC were lower than those in healthy individuals, and CRP levels were higher in patients with CRC than those in healthy individuals. Furthermore, the trauma of surgery and the postoperative stress reaction result in compromised nutritional status and increased inflammation. In our study, the levels of ALB and PA following surgery were significantly lower than those prior to surgery. The levels of CRP were significantly higher following surgery, which indicated that surgery induced inflammation. WD treatment following laparoscopic surgery in patients with CRC resulted in faster recovery of nutritional status and decreased inflammation, which indicated that WD promoted faster recovery of patients with CRC following surgery.

When patients undergo abdominal surgery, the digestive system stops working for several days, a phenomenon called postoperative ileus (POI). Postoperative ileus is common, painful, and results in significant discomfort. It is estimated that up to one-third of people who have undergone bowel surgery suffer from POI [37]. Failure of peristalsis results in accumulation of gastrointestinal secretions, leading to abdominal pain, symmetrical abdominal distension, anorexia, nausea or vomiting, and failure to pass stool or flatus [38]. Furthermore, failure of peristalsis is associated with increased patient morbidity, hospital costs, and 30-day readmission rates [25]. Therefore, postoperative gastrointestinal dysfunction seriously affects the rate of recovery of patients following surgery. In our study, WD treatment following surgery significantly promoted rapid recovery of gastrointestinal function in patients with CRC, resulting in a faster return to an oral diet. Faster recovery can reduce the incidence of complications, morbidity, and mortality following surgery and may promote comprehensive and rapid rehabilitation of patients with CRC.

To identify potential biomarkers and explain the mechanism of WD-related rapid recovery following laparoscopic surgery, changes in abundance of serum metabolites were monitored using an UPLC/Q-TOF-MS/MS-based metabolomic method. Twenty metabolites were identified as potential biomarkers in the MG, FTG, and WDG groups. These metabolites included LysoPC (16:0), LysoPC (18:1), LysoPC (18:0), LysoPC (18:2), LysoPC (22:6), LysoPE (16:0), 11-HETE, leucine, glutamine, 5-hydroxytryptamine, tryptophan, cystine, thromboxane B_2_, LysoPE (22:6), arachidonic acid, pyruvic acid, *α*-linolenic acid, palmitic acid, 9-HODE, and histidine. These metabolites are involved in arachidonic acid metabolism; alanine, aspartate, and glutamate metabolism; *α*-linolenic acid metabolism; pyruvate metabolism; histidine metabolism; and glycerophospholipid metabolism. The WDG and FTG profiles showed an apparent returning trend from that of MG, and some differences were observed between the WDG and FTG groups.

Among the 20 differentially abundant metabolites, mostly were lipids, including LysoPC (16:0), LysoPC (18:1), LysoPC (18:0), LysoPC (18:2), LysoPC (22:6), LysoPE (16:0), and LysoPE (22:6). Each lipid was significantly increased in the MG group compared with the HG group. Lysophosphatidylcholines (LysoPCs) are very sensitive to pathophysiological stimuli and are often used as biomarkers in metabolomic and lipidomic studies. Several studies have suggested that lysoPCs generated from phospholipase A_2_- (PLA_2_-) catalyzed hydrolysis of phosphatidylcholines (PCs) were involved in chronic inflammatory diseases, including tumor progression [39, 40]. In addition, abnormal lipid metabolism might increase the severity of inflammation [41]. Thus, elevated lysoPC and LysoPE levels may aggravate inflammation, resulting in cancer progression. However, after surgical resection of solid tumors combined with WD treatment, dysregulation of lysoPCs and LysoPEs was significantly reduced, which indicated that laparoscopic surgery combined with WD could influence glycerophospholipid metabolism, which may have contributed to rapid recovery of patients with CRC.

Arachidonic acid, 11-HETE, and thromboxane B_2_ in serum are products of arachidonic acid metabolism. Arachidonic acid is a major polyunsaturated fatty acid, is a precursor of many important eicosanoids (arachidonic acid-derived inflammatory mediators) in mammals, and is abundant at the sn-2 position of membrane phospholipids [42]. Arachidonic acid can be metabolized by cyclooxygenases (COX) [43], lipoxygenases (LOX) [44], or cytochrome P450s (CYP450) [45]. Cyclooxygenases convert arachidonic acid to prostaglandins (PGs) and thromboxanes (TXs), LOX convert arachidonic acid to leukotrienes (LT), and CYP450 and LOX produce “nonclassical” eicosanoids including arachidonic acid-derived hydroxyeicosatetraenoic acids (HETEs) and linoleic acid-derived hydroxyoctadecadienoic acids (HODEs) [46]. Development and progression of cancer is a multistep process regulated by many internal factors, including arachidonic acid-derived lipid mediators [47]. Clinical, animal, and cell-based studies have shown that the arachidonic acid metabolic pathway is activated and plays an important role in inflammation and tumorigenesis [48]. In our study, arachidonic acid and 11-HETE levels were higher in the MG group than those in the HG group, and TXB_2_ levels were significantly lower in the MG group than those in the HG group, which suggested an imbalance in arachidonic acid metabolism. Serum levels of arachidonic acid and 11-HETE were significantly lower in the FTG and WDG groups compared with those in the MG group, and TXB_2_ levels were significantly higher in the FTG and WDG groups than those in the MG group. Moreover, arachidonic acid levels in the WDG group were more similar to those in the HG group than those in the FTG group. In addition, linoleic acid-derived 9-HODE levels in the WDG group were more similar to those in the HG group than those in the FTG and MG group. These results indicated that laparoscopic surgery combined with WD could regulate the arachidonic acid and linoleic acid metabolism to promote rapid recovery of patients with CRC.

*α*-Linolenic acid is an n-3 polyunsaturated fatty acid (PUFA) and is one of two essential fatty acids required in the human diet [49]. Studies have shown that changes in PUFA metabolism occurred during colon cancer progression in human patients. In addition, reduced levels of linolenic acid have been observed in human colon adenomas and adenocarcinomas [50]. Some studies have found that linolenic acid reduced cell proliferation and induced apoptosis in CaCo-2 human colon adenocarcinoma cells [51]. In our study, linolenic acid levels were significantly lower in the MG group than those in the HG group, which were consistent with other studies. Furthermore, that levels of linolenic acid in the WDG group were significantly higher than those in the FTG and MG groups, and there was no difference between the FTG and MG groups. The results indicated that addition of WD may promote rapid recovery of patients with CRC by regulating α-linolenic acid metabolism.

Glutamine is a nonessential amino acid that is believed to become a “conditionally essential” amino acid during stress due to its importance in maintaining redox balance, its role in stimulating immune function, and its role as an energy source for enterocytes [52]. Glutamine can inhibit activation of NF-*κ*B, an important transcription factor which regulates inflammation, resulting in decreased inflammation [53]. Glutamine is consumed at high rates by many cancers and actively proliferating cells to support bioenergetics [54, 55]. Our results showed that glutamine levels were significantly decreased in the MG group compared to those in the HG group. However, glutamine levels were significantly increased in the WGD group compared to those in the MG group, which indicated that WD may promote rapid recovery of patients with CRC by regulating glutamine metabolism.

The phenomenon in which cancer cells produce energy to support cell growth and proliferation differently than normal cells is known as the “Warburg effect,” which is characterized by use of aerobic glycolysis as an energy source, even when oxygen is sufficient [56]. One of the main molecular mechanisms leading to the “Warburg effect” is mitochondrial dysfunction caused by dysfunctional pyruvate metabolism [57]. Pyruvate is a monocarboxylate molecule important in tumor metabolism [58]. In the mitochondria, pyruvate is oxidized by pyruvate dehydrogenase (PDH) to form acetyl-CoA, which is important in ATP synthesis. Pyruvate fuels mitochondrial oxygen consumption and reserves respiratory capacity. Increased mitochondrial metabolism has been shown to correlate with cell proliferation and aggressiveness in vitro [59]. In our study, pyruvate levels in the MG group were significantly higher than those in the HG group. Treatment with WD restored pyruvate to levels similar to those in the HG group. These results suggested that laparoscopic surgery combined with WD may promote rapid recovery in patients with CRC by regulating pyruvate metabolism and thereby affecting energy metabolism.

Previous studies have shown that urine concentrations of histidine, an essential amino acid, were significantly reduced in patients with CRC, as determined using CE-MS [60]. Moreover, a previous study showed that serum histidine levels were low in patients with pancreatic cancer [61]. Reduced histidine levels may have been due to higher activity of histidine decarboxylase (HDC) in these cancers, resulting in accelerated decarboxylation of histidine to histamine [62, 63]. In our study, serum levels of histidine were lower in the MG group than those in the HG group, which were consistent with previous studies. There were no significant differences in histidine levels between FTG and MG groups, but histidine levels in the WDG group were significantly higher than those in the MG group. These results suggested that WD therapy may promote rapid recovery of patients with CRC by regulating histidine metabolism.

Tryptophan metabolism is exploited by cancers to evade immune surveillance [64, 65]. Tryptophan is an essential amino acid and is metabolized through two metabolic pathways: the kynurenine pathway (KP) and the serotonin pathway. Approximately 95% of ingested tryptophan is metabolized via the KP [66]. Low levels of tryptophan and increased concentrations of its degradation product, kynurenine, may be directly involved in reduced T-cell responsiveness to antigenic stimulation in cancer [67]. In addition, low levels of tryptophan in the local microenvironment have been shown to activate stress-response pathways, including GCN2 kinase and mTOR [68, 69]. Several studies have reported reduced tryptophan levels and increased KP metabolites in patients with colon cancer [67, 70], which were consistent with our findings. After surgery combined with WD treatment, tryptophan levels were similar to those in the HG group. These results suggested that surgery combined with WD may promote rapid recovery of patients with CRC by regulating tryptophan metabolism and thereby regulating the immune function.

Serotonin (5-HT) is a monoamine hormone and neurotransmitter. Serotonin is an important gastrointestinal regulator and plays an important role in regulating gastrointestinal physiology, including peristalsis and motility, secretion, and absorption of nutrients [71, 72]. Specifically, 5-HT can activate peristaltic reflexes, resulting in gastrointestinal motility, and it can also regulate intestinal inflammation. In our study, we found that 5-HT levels were not significantly different between the FTG and MG groups but were significantly increased in the WDG group. These results suggested that WD may promote recovery of gastrointestinal function by promoting gastrointestinal motility after surgery, resulting in rapid recovery of patients with CRC.

Recent studies have reported that reduction of leucine levels inhibited proliferation of ER^+^ (breast cancers that express estrogen receptors) breast cancer cells, whereas increased leucine levels enhanced proliferation of ER^+^ breast cancer cells. In addition, a previous study showed that a diet deficient in leucine may be beneficial for patients with ER^+^ breast cancer [73]. In our study, we found that leucine levels in the MG group were significantly higher than those in the HG group, which may have been associated with proliferation of CRC cells. In contrast, leucine levels in the FTG and WDG groups were significantly lower than those in the MG group. Moreover, leucine levels in the WDG group were similar to those in the HG group, but leucine levels in the FTG group did not approach those in the HG group. These results suggested that surgery combined with WD may promote rapid recovery of patients with CRC by regulating leucine levels, resulting in reduced proliferation of CRC cells.

Palmitic acid is a saturated fatty acid found in animals, plants, and human milk fat. Palmitic acid has been shown to affect cell proliferation and tumor development in vitro and in vivo [74, 75]. A previous study showed that CRC risk was positively associated with erythrocyte membrane palmitic acid content [76]. The levels of palmitic acid in the MG group were significantly higher than those in the HG group, which agreed with results of previous studies. Surgery combined with WD significantly reduced palmitic acid levels, which suggested that surgery combined with WD may promote rapid recovery of patients with CRC by regulating membrane palmitic acid composition.

## 5. Conclusion

In summary, serum biochemistry, detection of clinical gastrointestinal function, and metabolomics strategies were integrated to explore the possible mechanisms of action of laparoscopic surgery combined with WD in rapid rehabilitation of patients with CRC. The results suggested that the therapeutic mechanism of laparoscopic surgery combined with WD may be related to regulation of nutritional status, inflammation, immune function, energy, and gastrointestinal function in patients with CRC. This study also highlighted the ability of WD to interact with multiple targets to induce synergistic effects. Our study indicated that WD is a potential therapeutic agent to promote recovery following resection of CRC tumors.

But the analysis of the metabolites is based on nontargeted metabolomics and statistical analysis, so 11 of the identified metabolites were validated with the commercially available reference substances. Specific details are shown in [Supplementary-material supplementary-material-1] and Figures [Supplementary-material supplementary-material-1]–[Supplementary-material supplementary-material-1]. In addition, our next step will continue to use the targeted approaches to further validate our above findings.

## Figures and Tables

**Table 1:** Demographic information of research population.

Variables	Fundamental rental treatment group, FTG (*n* = 30)	Wuda granule group, WDG (*n* = 30)	Healthy group, HG (*n* = 30)
Gender (male/female)	11/19	13/17	16/14
Age (years)	60.77 ± 9.58	61.53 ± 10.02	51.9 ± 8.03
Laparoscopic colorectal cancer surgery(yes/no)	Yes	Yes	No
Left hemicolectomy	2	4	
Right hemicolectomy	9	9	
Rectectomy	5	7	
Sigmoidectomy	12	7	
Transverse colectomy	1	2	
Ascending colon resection	1	1	

The age of the three groups is expressed in the form of mean ± SD.

**Table 2:** Representative serum metabolites' RSDs of the retention time and peak area in positive and negative ion modes (*n* = 6).

Ion model	Metabolite	Formula	Adduct	Extraction mass (Da)	RSD% RT (min)	RSD% area
Positive	5-Hydroxytryptamine	C_10_H_12_N_2_O	[M+H]^+^	1.06	1.06	8.49
	Tryptophan	C_11_H_12_N_2_O_2_	[M+H]^+^	0.24	0.24	3.76
	LysoPC (18:2)	C_26_H_50_O_7_NP	[M+H]^+^	0.05	0.05	7.25
	LysoPC (16:0)	C_24_H_50_O_7_NP	[M+H]^+^	0.06	0.06	5.98
	LysoPC (18:1)	C_26_H_52_O_7_NP	[M+H]^+^	0.05	0.05	5.78
	LysoPC (18:0)	C_26_H_54_O_7_NP	[M+H]^+^	0.05	0.05	3.27

Negative	Gluconic acid	C_6_H_12_O_7_	[M–H]^–^	195.05	0.77	10.66
	Glycocholic acid	C_26_H_43_NO_6_	[M–H]^–^	464.30	0.15	3.74
	Glycoursodeoxycholic acid	C_20_H_32_O_2_	[M–H]^–^	448.31	0.10	6.85
	LysoPE (22:6)	C_26_H_43_NO_5_	[M–H]^–^	524.28	0.07	3.14
	Arachidonic acid	C_20_H_32_O_2_	[M–H]^–^	303.23	0.03	5.93
	Palmitic acid	C_16_H_32_O_2_	[M–H]^–^	255.23	0.04	8.37

*Note.* RSD: relative standard deviation.

**Table 3:** Identification results of varying ions and their change trend.

No.	Metabolite	VIP	RT (min)	Formula	Adduct	Calculated	Trend			Related pathway
							MG/HG	FTG/MG	WDG/MG	
1☆	LysoPC (16:0)	11.93	10.11	C_24_H_50_O_7_NP	[M+H]^+^	496.3398	↑^∗∗∗^	↓^###^	↓^&&&^	Glycerophospholipid metabolism
2	LysoPC (18:1)	9.57	10.29	C_26_H_52_O_7_NP	[M+H]^+^	522.3554	↑^∗∗∗^	↓^###^	↓^&&&^	Glycerophospholipid metabolism
3☆	LysoPC (18:0)	9.41	11.25	C_26_H_54_O_7_NP	[M+H]^+^	524.3711	↑^∗∗∗^	↓^###^	↓^&&&^	Glycerophospholipid metabolism
4	LysoPC (18:2)	8.33	9.60	C_26_H_50_O_7_NP	[M+H]^+^	520.3398	↑^∗∗∗^	↓^###^	↓^&&&^	Glycerophospholipid metabolism
5	LysoPC (22:6)	6.61	9.61	C_30_H_50_O_7_NP	[M+H]^+^	568.3398	↑^∗∗∗^	↓^##^	↓^&&&^	Glycerophospholipid metabolism
6	LysoPE (16:0)	5.29	9.92	C_21_H_44_O_7_NP	[M+H]^+^	454.2928	↑^∗∗∗^	↓^###^	↓^&&&^	Glycerophospholipid metabolism
7	11-HETE	3.22	9.75	C_20_H_32_O_3_	[M+H]^+^	321.2424	↑^∗∗∗^	↓^###^	↓^&&&^	Arachidonic acid metabolism
8☆	Leucine	2.98	1.62	C_6_H_13_NO_2_	[M+H]^+^	132.1019	↑^∗∗∗^	↓^#^	↓^&&&^	Other
9☆	Glutamine	2.45	0.66	C_5_H_10_N_2_O_3_	[M+H]^+^	147.0764	↓^∗∗∗^	↑^###^	↑^&&&^	Alanine, aspartate, and glutamate metabolism
10☆	5-Hydroxytryptamine	2.11	1.28	C_10_H_12_N_2_O	[M+H]^+^	177.1022	↓^∗∗^	↑	↑^&&&^	Tryptophan metabolism
11☆	Tryptophan	2.02	3.59	C_11_H_12_N_2_O_2_	[M+H]^+^	205.0972	↓^∗∗∗^	↑^###^	↑^&&&^	Tryptophan metabolism
12	Cystine	1.91	1.59	C_6_H_12_N_2_O_4_S_2_	[M+H]^+^	241.0311	↑^∗∗^	↓^#^	↓	Other
13	Thromboxane B2	1.74	4.14	C_20_H_34_O_6_	[M+H]^+^	371.2428	↓^∗∗∗^	↑^###^	↑^&&&^	Arachidonic acid metabolism
14	LysoPE (22:6)	9.21	9.59	C_27_H_44_O_7_NP	[M–H]^–^	524.2783	↑^∗∗∗^	↓^###^	↓^&&&^	Glycerophospholipid metabolism
15☆	Arachidonic acid	5.12	12.50	C_20_H_32_O_2_	[M–H]^–^	303.2331	↑^∗∗∗^	↓^#^	↓^&&&^	Arachidonic acid metabolism
16☆	Pyruvic acid	3.98	0.87	C_3_H_4_O_3_	[M–H]^–^	87.0088	↑^∗∗∗^	↓^###^	↓^&&&^	Pyruvate metabolism
17☆	*α*-Linolenic acid	3.16	12.04	C_18_H_30_O_2_	[M–H]^–^	277.2173	↓^∗∗∗^	↓	↑^&^	*α*-Linolenic acid metabolism
18☆	Palmitic acid	2.64	13.13	C_16_H_32_O_2_	[M–H]^–^	255.2329	↑^∗∗∗^	↓^##^	↓^&&^	Other
19	9-HODE	2.37	10.21	C_18_H_32_O_3_	[M–H]^–^	295.2279	↑^∗∗∗^	↓^###^	↓^&&&^	Linoleic acid metabolism
20☆	Histidine	1.85	0.62	C_6_H_9_N_3_O_2_	[M–H]^–^	154.0622	↓	↓	↑^&&^	Histidine metabolism

*Note.* ☆: confirmed with the reference substances. ↑: the compound is upregulated. ↓: the compound is downregulated. Represents the changes in MG compared with HG, ^*∗*^*p* < 0.05, ^*∗∗*^*p* < 0.01, *p* < 0.001; represents the changes in FTG compared with MG, ^#^*p* < 0.05, ^##^*p* < 0.01, ^###^*p* < 0.001; and represents the changes in WDG compared with MG, ^&^*p* < 0.05, ^&&^*p* < 0.01, ^&&&^*p* < 0.001.

**Table 4:** Metabolic pathway enrichment analysis results of using MetaboAnalyst software.

No.	Metabolic pathway	Total	Raw *p*	−Log(*p*)	Impact	No. in Figure 7
1	Arachidonic acid metabolism	62	0.042429	3.1599	0.2255	a
2	Alanine, aspartate, and glutamate metabolism	24	0.006952	4.9687	0.20703	b
3	*α*-Linolenic acid metabolism	29	0.14613	1.9233	0.20335	c
4	Pyruvate metabolism	32	0.16006	1.8322	0.18254	d
5	Histidine metabolism	44	0.21372	1.5431	0.13988	e

## Data Availability

The data used to support the findings of this study are available from the corresponding author upon request.

## Abbreviations

UPLC/Q-TOF-MS/MS: Ultraperformance liquid chromatography with electrospray ionization quadrupole time-of-flight tandem mass spectrometry
WD: Wuda granule
PCA: Principal component analysis
OPLS-DA: Orthogonal partial least square-discriminant analysis
VIP: Variable importance in the projection
QC: Quality control
RSD: Relative standard deviation
ERAS: Enhanced recovery after surgery
CRC: Colorectal cancer
POI: Postoperative ileus
TCM: Traditional Chinese medicine
HG: Healthy group
MG: Model group
FTG: Fundamental treatment group
WDG: Wuda granule group
TIC: Total ion chromatogram
PA: Prealbumin
ALB: Albumin
CRP: C-reactive protein
LysoPC: Lysophosphatidylcholine
LysoPE: Lysophosphatidylethanolamine
KP: Kynurenine pathway
PUFA: Polyunsaturated fatty acid.

## Supplementary Materials

Supplementary Materials Table S1: the detailed information for the 11 reference substances uesd in this study. Figure S1: the TICs of the superposition of the QC samples in the positive and negative ion mode. A, in positive ion modes; B, in negative ion modes. Figure S2: the EIC and MS/MS spectra for LysoPC (16:0) from the reference substance (A and C) and serum sample (B and D). Figure S3: the EIC and MS/MS spectra for LysoPC (18:0) from the reference substance (A and C) and serum sample (B and D). Figure S4: the EIC and MS/MS spectra for leucine from the reference substance (A and C) and serum sample (B and D). Figure S5: the EIC and MS/MS spectra for glutamine from the reference substance (A and C) and serum sample (B and D). Figure S6: the EIC and MS/MS spectra for 5-hydroxytryptamine from the reference substance (A and C) and serum sample (B and D). Figure S7: the EIC and MS/MS spectra for tryptophan from the reference substance (A and C) and serum sample (B and D). Figure S8: the EIC and MS/MS spectra for arachidonic acid from the reference substance (A and C) and serum sample (B and D). Figure S9: the EIC and MS/MS spectra for pyruvic acid from the reference substance (A and C) and serum sample (B and D). Figure S10: the EIC and MS/MS spectra for α-linolenic acid from the reference substance (A and C) and serum sample (B and D). Figure S11: the EIC and MS/MS spectra for palmitic acid from the reference substance (A and C) and serum sample (B and D). Figure S12: the EIC and MS/MS spectra for histidine from the reference substance (A and C) and serum sample (B and D).

## References

[1] Rezapour S., Bahrami T., Hashemzadeh S. (2016). STC1 and NF-kappaB p65 (Rel A) is constitutively activated in colorectal cancer. *Clinical Laboratory*.

[2] Lech G. (2016). Colorectal cancer tumour markers and biomarkers: recent therapeutic advances. *World Journal of Gastroenterology*.

[3] Brody H. (2015). Colorectal cancer. *Nature*.

[4] Weinberg B. A., Marshall J. L., Salem M. E. (2017). The growing challenge of young adults with colorectal cancer. *Oncology (Williston Park)*.

[5] Brenner H., Kloor M., Pox C. P. (2014). Colorectal cancer. *Lancet*.

[6] Guo P., Huang Z. L., Yu P., Li K. (2012). Trends in cancer mortality in China: an update. *Annals of Oncology*.

[7] Kuipers E. J., Grady W. M., Lieberman D. (2015). Colorectal cancer. *Nature Review Disease Primers*.

[8] Gustafsson U. O., Scott M. J., Schwenk W. (2012). Guidelines for perioperative care in elective colonic surgery: Enhanced Recovery after Surgery (ERAS) Society recommendations. *Clinical Nutrition*.

[9] Nygren J., Thacker J., Carli F. (2012). Guidelines for perioperative care in elective rectal/pelvic surgery: Enhanced Recovery after Surgery (ERAS) Society recommendations. *Clinical Nutrition*.

[10] Tabrizian P., Franssen B., Jibara G. (2014). Cytoreductive surgery with or without hyperthermic intraperitoneal chemotherapy in patients with peritoneal hepatocellular carcinoma. *Journal of Surgical Oncology*.

[11] Arjona-Sanchez A., Muñoz-Casares C., Ortega-Salas R., Casado-Adam A., Sanchez-Hidalgo J. M., Rufián-Peña S. (2014). Long-term survival with peritoneal mucinous carcinomatosis from intraductal mucinous papillary pancreatic carcinoma treated with complete cytoreduction and hyperthermic intraperitoneal chemotherapy. *International Journal of Hyperthermia*.

[12] Boyce M. J., Baisley K. J., Warrington S. J. (2012). Pharmacokinetic interaction between domperidone and ketoconazole leads to QT prolongation in healthy volunteers: a randomized, placebo-controlled, double-blind, crossover study. *British Journal of Clinical Pharmacology*.

[13] Cui Y., Chen H., Qi L., Zu X., Li Y. (2016). Effect of alvimopan on accelerates gastrointestinal recovery after radical cystectomy: a systematic review and meta-analysis. *International Journal of Surgery*.

[14] Sultan S., Coles B., Dahm P. (2017). Alvimopan for recovery of bowel function after radical cystectomy. *Cochrane Database of Systematic Reviews*.

[15] Schmidt J., Stoffels B., Nazir A., Dehaven-Hudkins D. L., Bauer A. J. (2008). Alvimopan and COX-2 inhibition reverse opioid and inflammatory components of postoperative ileus. *Neurogastroenterology & Motility*.

[16] Jiang Z., Cao L.-X., Liu B. (2017). Effects of Chinese herbal medicine Xiangbin prescription on gastrointestinal motility. *World Journal of Gastroenterology*.

[17] Wen S.-L., Feng X., Chen Z.-Q., Xiao J., Zhang W.-X. (2016). Effect of XiangBin granules on post-operative gastrointestinal function and brain-gut peptides after transabdominal gynecological surgery. *European Journal of Obstetrics & Gynecology and Reproductive Biology*.

[18] Gan H., Lin J., Jiang Z., Chen Q., Cao L., Chen Z. (2018). Xiangbin prescription for the recovery of gastrointestinal function after abdominal surgery (the XBPRS trial): study protocol for a randomized controlled trial. *Trials*.

[19] Xu F. F., Qi R., Jiang S. W. (2017). Simultaneous determination of ten compounds in Xiangbin Fang by LC-MRM-MS. *China Journal of Traditional Chinese Medicine and Pharmacy*.

[20] Jiang S. W., Zheng Z. L., Xu F. F. (2019). Simultaneous determination of four alkaloid components in Xiangbin decoction by UPLC-QqQ-MS/MS. *Chinese Journal of Experimental Traditional Medical Formulae*.

[21] Kaddurah-Daouk R., Kristal B. S., Weinshilboum R. M. (2008). Metabolomics: a global biochemical approach to drug response and disease. *Annual Review of Pharmacology and Toxicology*.

[22] Wang M., Chen L., Liu D., Chen H., Tang D.-D., Zhao Y.-Y. (2017). Metabolomics highlights pharmacological bioactivity and biochemical mechanism of traditional Chinese medicine. *Chemico-Biological Interactions*.

[23] Cao D., Xu C., Xue Y. (2018). The therapeutic effect of Ilex pubescens extract on blood stasis model rats according to serum metabolomics. *Journal of Ethnopharmacology*.

[24] Greene F. L., Kercher K. W., Nelson H., Teigland C. M., Boller A.-M. (2007). Minimal access cancer management. *CA: A Cancer Journal for Clinicians*.

[25] Li L. T., Mills W. L., White D. L. (2013). Causes and prevalence of unplanned readmissions after colorectal surgery: a systematic review and meta-analysis. *Journal of the American Geriatrics Society*.

[26] Truong A., Hanna M. H., Moghadamyeghaneh Z., Stamos M. J. (2016). Implications of preoperative hypoalbuminemia in colorectal surgery. *World Journal of Gastrointestinal Surgery*.

[27] Liu Z., Jin K., Guo M. (2016). Prognostic value of the CRP/alb ratio, a novel inflammation-based score in pancreatic cancer. *Annals of Surgical Oncology*.

[28] Takaaki F., Toshinaga S., Hiroki M. (2012). Serum albumin is superior to prealbumin for predicting short-term recurrence in patients with operable colorectal cancer. *Nutrition & Cancer*.

[29] McIntosh E. N., Laurent L. L. (1983). Nutritional assessment of the hospitalized patient. *American Family Physician*.

[30] Delmore G. (1997). Assessment of nutritional status in cancer patients: widely neglected?. *Supportive Care in Cancer*.

[31] McDermott F. D., Heeney A., Kelly M. E., Steele R. J., Carlson G. L., Winter D. C. (2015). Systematic review of preoperative, intraoperative and postoperative risk factors for colorectal anastomotic leaks. *British Journal of Surgery*.

[32] Maykel J. A. (2014). *Perioperative Nutrition Support in Colorectal Surgery*.

[33] Digant G., Lis C. G., Joel G., Grutsch J. F., Vashi P. G., Lammersfeld C. A. (2006). Malnutrition was associated with poor quality of life in colorectal cancer: a retrospective analysis. *Journal of Clinical Epidemiology*.

[34] Schwegler I., Von H. A. J. (2010). Nutritional risk is a clinical predictor of postoperative mortality and morbidity in surgery for colorectal cancer. *British Journal of Surgery*.

[35] Balkwill F., Mantovani A. (2001). Inflammation and cancer: back to Virchow?. *The Lancet*.

[36] Moore M. M., Chua W., Charles K. A., Clarke S. J. (2010). Inflammation and cancer: causes and consequences. *Clinical Pharmacology & Therapeutics*.

[37] Vaneesha S., Georgia H., Rachel P. (2015). *Chewing Gum for Postoperative Recovery of Gastrointestinal Function*.

[38] Bragg D., El-Sharkawy A. M., Psaltis E., Maxwell-Armstrong C. A., Lobo D. N. (2015). Postoperative ileus: recent developments in pathophysiology and management. *Clinical Nutrition*.

[39] Linkous A. G., Yazlovitskaya E. M., Hallahan D. E. (2010). Cytosolic phospholipase A2 and lysophospholipids in tumor angiogenesis. *Journal of the National Cancer Institute*.

[40] Zhang T., Wu X., Yin M. (2012). Discrimination between malignant and benign ovarian tumors by plasma metabolomic profiling using ultra performance liquid chromatography/mass spectrometry. *Clinica Chimica Acta*.

[41] Hung N. D., Sok D.-E., Kim M. R. (2012). Prevention of 1-palmitoyl lysophosphatidylcholine-induced inflammation by polyunsaturated acyl lysophosphatidylcholine. *Inflammation Research*.

[42] Yagami T., Yamamoto Y., Koma H. (2018). Physiological and pathological roles of 15-deoxy-Δ12,14-prostaglandin J2 in the central nervous system and neurological diseases. *Molecular Neurobiology*.

[43] Willenberg I., Meschede A. K., Schebb N. H. (2015). Determining cyclooxygenase-2 activity in three different test systems utilizing online-solid phase extraction-liquid chromatography-mass spectrometry for parallel quantification of prostaglandin E2, D2 and thromboxane B2. *Journal of Chromatography A*.

[44] Lehmann C., Homann J., Ball A.-K. (2015). Lipoxin and resolvin biosynthesis is dependent on 5-lipoxygenase activating protein. *The FASEB Journal*.

[45] Fleming I. (2011). Cytochrome P450-dependent eicosanoid production and crosstalk. *Current Opinion in Lipidology*.

[46] Wolfer A. M., Scott A. J., Rueb C. (2017). Longitudinal analysis of serum oxylipin profile as a novel descriptor of the inflammatory response to surgery. *Journal of Translational Medicine*.

[47] Grizzi F., Di Ieva A., Russo C. (2006). Cancer initiation and progression: an unsimplifiable complexity. *Theoretical Biology and Medical Modelling*.

[48] Wang D., Dubois R. N. (2010). Eicosanoids and cancer. *Nature Reviews Cancer*.

[49] Calviello G., Serini S., Piccioni E. (2007). n-3 polyunsaturated fatty acids and the prevention of colorectal cancer: molecular mechanisms involved. *Current Medicinal Chemistry*.

[50] Fernandez-Banares F., Esteve M., Navarro E. (1996). Changes of the mucosal n3 and n6 fatty acid status occur early in the colorectal adenoma-carcinoma sequence. *Gut*.

[51] Bommareddy A., Zhang X. Y., Kaushik R. S., Dwivedi C. (2010). Effects of components present in flaxseed on human colon adenocarcinoma Caco-2 cells: possible mechanisms of flaxseed on colon cancer development in animals. *Drug Discovery and Therapeutics*.

[52] Marian M. J. (2017). Dietary supplements commonly used by cancer survivors: are there any benefits?. *Nutrition in Clinical Practice*.

[53] Beutheu S., Ouelaa W., Guérin C. (2014). Glutamine supplementation, but not combined glutamine and arginine supplementation, improves gut barrier function during chemotherapy-induced intestinal mucositis in rats. *Clinical Nutrition*.

[54] Cluntun A. A., Lukey M. J., Cerione R. A., Locasale J. W. (2017). Glutamine metabolism in cancer: understanding the heterogeneity. *Trends in Cancer*.

[55] Daye D., Wellen K. E. (2012). Metabolic reprogramming in cancer: unraveling the role of glutamine in tumorigenesis. *Seminars in Cell & Developmental Biology*.

[56] Vander H. M., Cantley L. C., Thompson C. B. (2009). Understanding the Warburg effect: the metabolic requirements of cell proliferation. *Science*.

[57] Diaz-Ruiz R., Rigoulet M., Devin A. (2011). The Warburg and Crabtree effects: on the origin of cancer cell energy metabolism and of yeast glucose repression. *Biochimica Biophysica Acta*.

[58] Martinez-Outschoorn U. E., Peiris-Pagés M., Pestell R. G., Sotgia F., Lisanti M. P. (2017). Cancer metabolism: a therapeutic perspective. *Nature Reviews Clinical Oncology*.

[59] Diers A. R., Broniowska K. A., Chang C.-F., Hogg N. (2012). Pyruvate fuels mitochondrial respiration and proliferation of breast cancer cells: effect of monocarboxylate transporter inhibition. *Biochemical Journal*.

[60] Chen J. L., Fan J., Yan L. S. (2012). Urine metabolite profiling of human colorectal cancer by capillary electrophoresis mass spectrometry based on MRB. *Gastroenterology Research and Practice*.

[61] Fukutake N., Ueno M., Hiraoka N. (2015). A novel multivariate index for pancreatic cancer detection based on the plasma free amino acid profile. *PLOS One*.

[62] Qiu Y., Cai G., Su M. (2010). Urinary metabonomic study on colorectal cancer. *Journal of Proteome Research*.

[63] Uchiyama K., Yagi N., Mizushima K. (2017). Serum metabolomics analysis for early detection of colorectal cancer. *Journal of Gastroenterology*.

[64] Prendergast G. C., Cancer (2011). Why tumours eat tryptophan. *Nature*.

[65] Löb S., Königsrainer A., Rammensee H.-G., Opelz G., Terness P. (2009). Inhibitors of indoleamine-2,3-dioxygenase for cancer therapy: can we see the wood for the trees?. *Nature Reviews Cancer*.

[66] Peters J. C. (1991). Tryptophan nutrition and metabolism: an overview. *Advances in Experimental Medicine and Biology*.

[67] Engin A. B., Karahalil B., Karakaya A. E., Engin A. (2015). Helicobacter pylori and serum kynurenine-tryptophan ratio in patients with colorectal cancer. *World Journal of Gastroenterology*.

[68] Fougeray S., Mami I., Bertho G., Beaune P., Thervet E., Pallet N. (2012). Tryptophan depletion and the kinase GCN2 mediate IFN-γ-Induced autophagy. *The Journal of Immunology*.

[69] Metz R., Rust S., Duhadaway J. B. (2012). IDO inhibits a tryptophan sufficiency signal that stimulates mTOR: a novel IDO effector pathway targeted by D-1-methyl-tryptophan. *Oncoimmunology*.

[70] Liu X., Shin N., Koblish H. K. (2010). Selective inhibition of IDO1 effectively regulates mediators of antitumor immunity. *Blood*.

[71] Layunta E., Latorre E., Forcén R. (2018). NOD1 downregulates intestinal serotonin transporter and interacts with other pattern recognition receptors. *Journal of Cellular Physiology*.

[72] Mawe G. M., Hoffman J. M. (2013). Serotonin signalling in the gut-functions, dysfunctions and therapeutic targets. *Nature Reviews Gastroenterology & Hepatology*.

[73] Saito Y., Li L., Coyaud E. (2019). LLGL2 rescues nutrient stress by promoting leucine uptake in ER+ breast cancer. *Nature*.

[74] Hardy S., Langelier Y., Prentki M. (2000). Oleate activates phosphatidylinositol 3-kinase and promotes proliferation and reduces apoptosis of MDA-MB-231 breast cancer cells, whereas palmitate has opposite effects. *Cancer Research*.

[75] Anna R., Laura Z., Lucia S. (2013). Influence of fatty acid-free diet on mammary tumor development and growth rate in HER-2/Neu transgenic mice. *Journal of Cellular Physiology*.

[76] Kiyonori K., Kenji W., Kaoru H. (2006). Risk of colorectal cancer is linked to erythrocyte compositions of fatty acids as biomarkers for dietary intakes of fish, fat, and fatty acids. *Cancer Epidemiology, Biomarkers & Prevention*.

