# Supplementary material for: Metabolomics Analysis of Laparoscopic Surgery Combined with Wuda Granule to Promote Rapid Recovery of Patients with Colorectal Cancer Using UPLC/Q-TOF-MS/MS
Source: Evid Based Complement Alternat Med. 2020 Feb 13;2020:5068268. doi: 10.1155/2020/5068268 (PMC7040410; doi:10.1155/2020/5068268)
Supplement: Supplementary Materials — Table S1: the detailed information for the 11 reference substances uesd in this study. Figure S1: the TICs of the superposition of the QC samples in the positive and negative ion mode. A, in positive ion modes; B, in negative ion modes. Figure S2: the EIC and MS/MS spectra for LysoPC (16:0) from the reference substance (A and C) and serum sample (B and D). Figure S3: the EIC and MS/MS spectra for LysoPC (18:0) from the reference substance (A and C) and serum sample (B and D). Figure S4: the EIC and MS/MS spectra for leucine from the reference substance (A and C) and serum sample (B and D). Figure S5: the EIC and MS/MS spectra for glutamine from the reference substance (A and C) and serum sample (B and D). Figure S6: the EIC and MS/MS spectra for 5-hydroxytryptamine from the reference substance (A and C) and serum sample (B and D). Figure S7: the EIC and MS/MS spectra for tryptophan from the reference substance (A and C) and serum sample (B and D). Figure S8: the EIC and MS/MS spectra for arachidonic acid from the reference substance (A and C) and serum sample (B and D). Figure S9: the EIC and MS/MS spectra for pyruvic acid from the reference substance (A and C) and serum sample (B and D). Figure S10: the EIC and MS/MS spectra for α-linolenic acid from the reference substance (A and C) and serum sample (B and D). Figure S11: the EIC and MS/MS spectra for palmitic acid from the reference substance (A and C) and serum sample (B and D). Figure S12: the EIC and MS/MS spectra for histidine from the reference substance (A and C) and serum sample (B and D). [file 5068268.f1.pdf]

Supplementary Materials

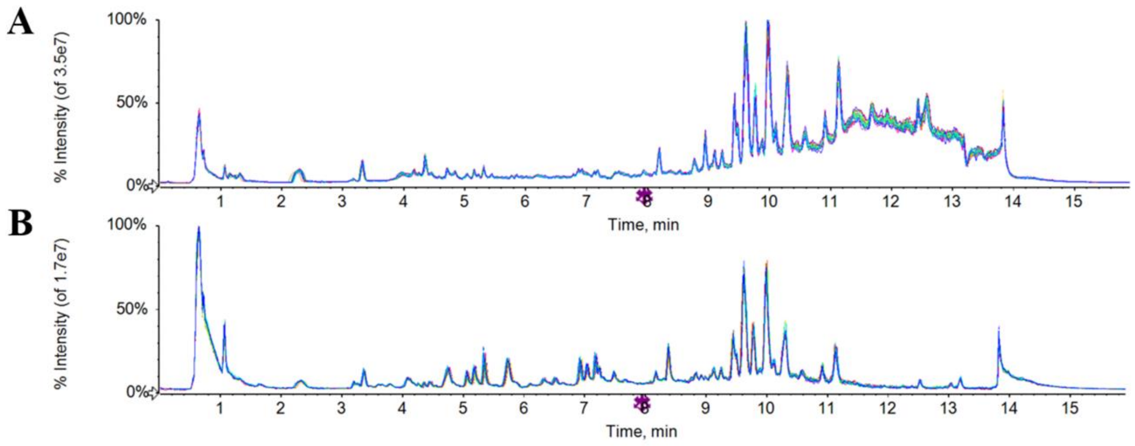

Figure S1: The TICs of the superposition of the QC samples in the positive and negative ion mode. A, in positive ion modes; B, in negative ion modes.

Table S1: The detailed information for the 11 reference substances uesd in this study.

Name	CAS No.	Lot No.	Manufacturer
Leucine	61-90-5	S10D9I76995	Shanghai yuanye Bio-Technology Co., Ltd
Glutamine	56-85-9	011M01991V	Shanghai yuanye Bio-Technology Co., Ltd
Tryptophan	73-22-3	S02D7I26049	Shanghai yuanye Bio-Technology Co., Ltd
Hisdine	71-00-1	Z19A9H59384	Shanghai yuanye Bio-Technology Co., Ltd
Palmitic acid	57-10-3	M20N6K6269	Shanghai yuanye Bio-Technology Co., Ltd
Pyruvic acid	127-17-3	H08S6Q3079	Shanghai yuanye Bio-Technology Co., Ltd
LysoPC (16:0)	17364-16-8	B1911189	Shanghai Aladdin Bio-Chem Technology Co., LTD
LysoPC (18:0)	19420-57-6	I1730026	Shanghai Aladdin Bio-Chem Technology Co., LTD
α-Linolenic acid	463-40-1	Q29J10Q79787	Shanghai yuanye Bio-Technology Co., Ltd
Arachidonic acid	506-32-1	Q18J10Q91289	Shanghai yuanye Bio-Technology Co., Ltd
Serotonin	50-67-9	BCCB5452	Sigma-aldrich

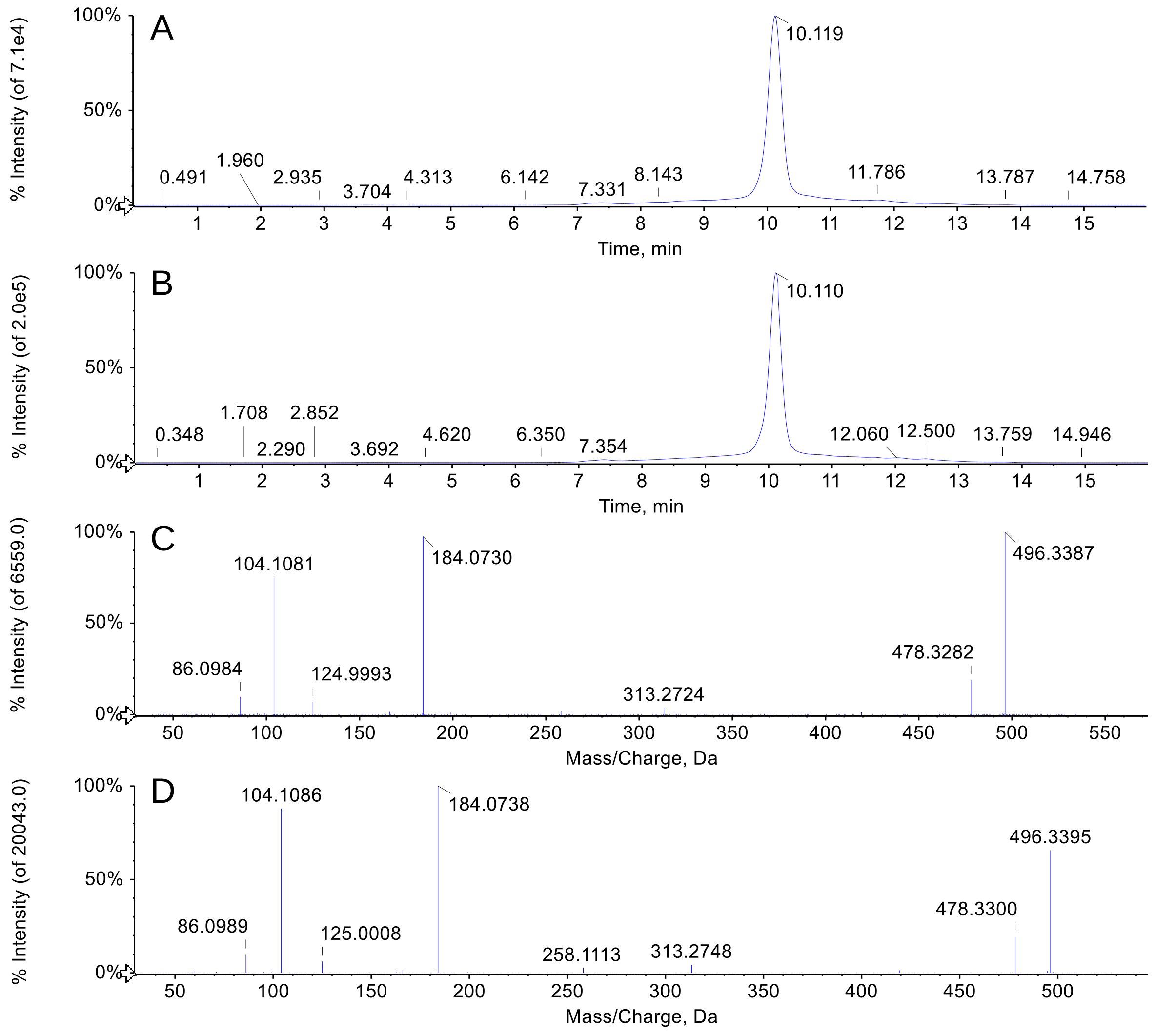

Fig S2: The EIC and MS/MS spectra for **LysoPC (16:0)** from the reference substance (A and C) and serum sample (B and D)

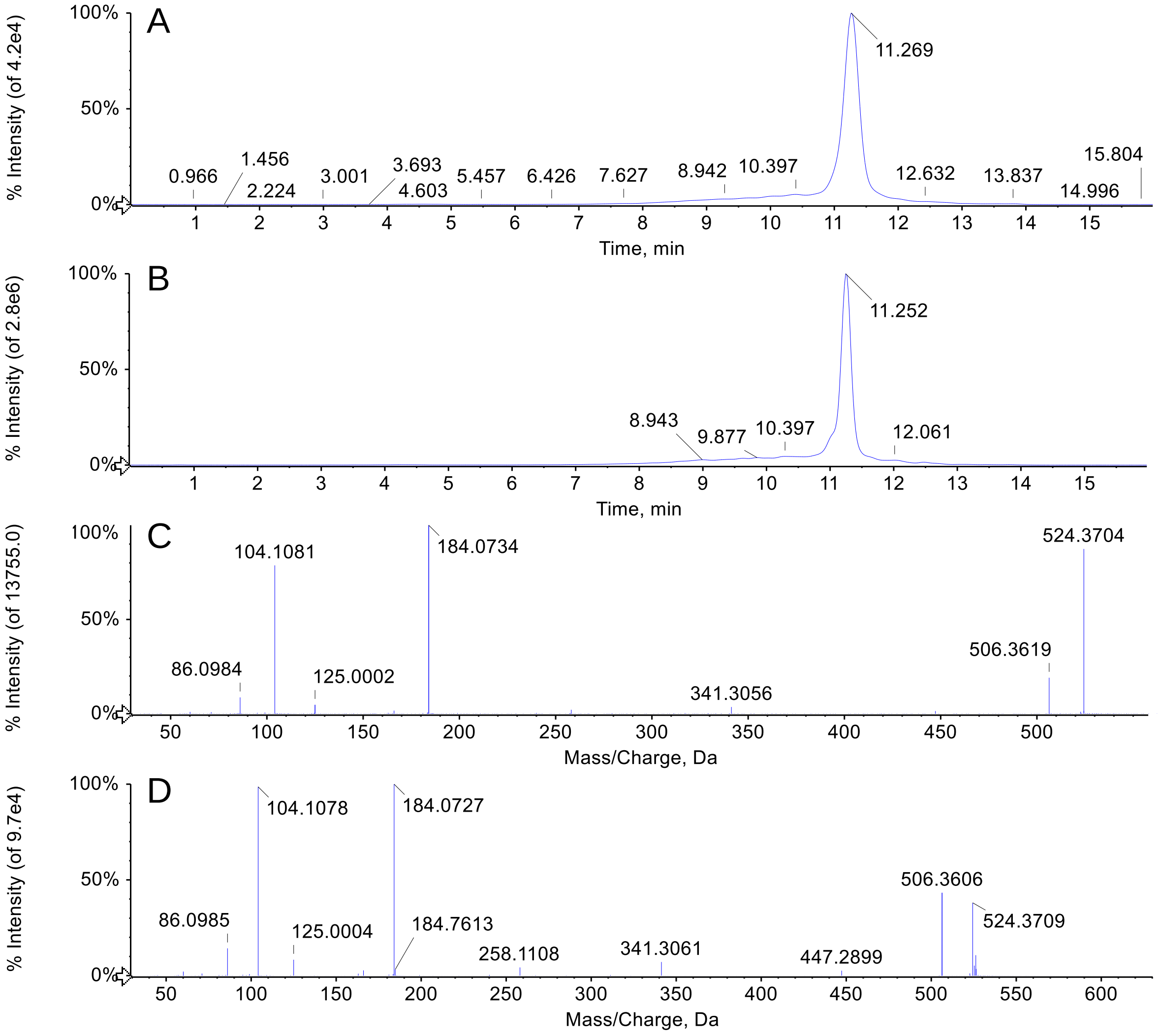

Fig S3: The EIC and MS/MS spectra for **LysoPC (18:0)** from the reference substance (A and C) and serum sample (B and D)

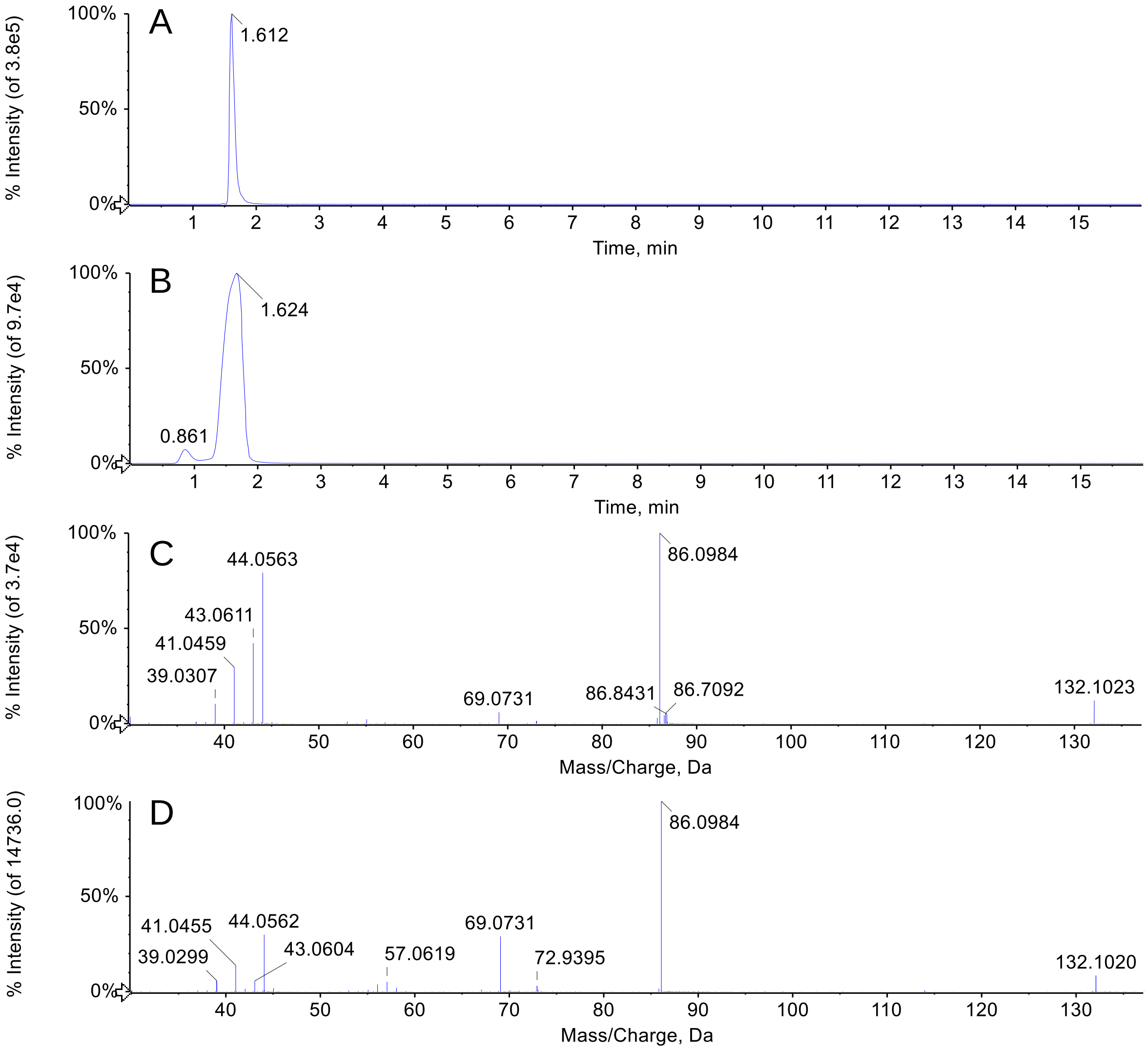

Fig S4: The EIC and MS/MS spectra for **leucine** from the reference substance (A and C) and serum sample (B and D)

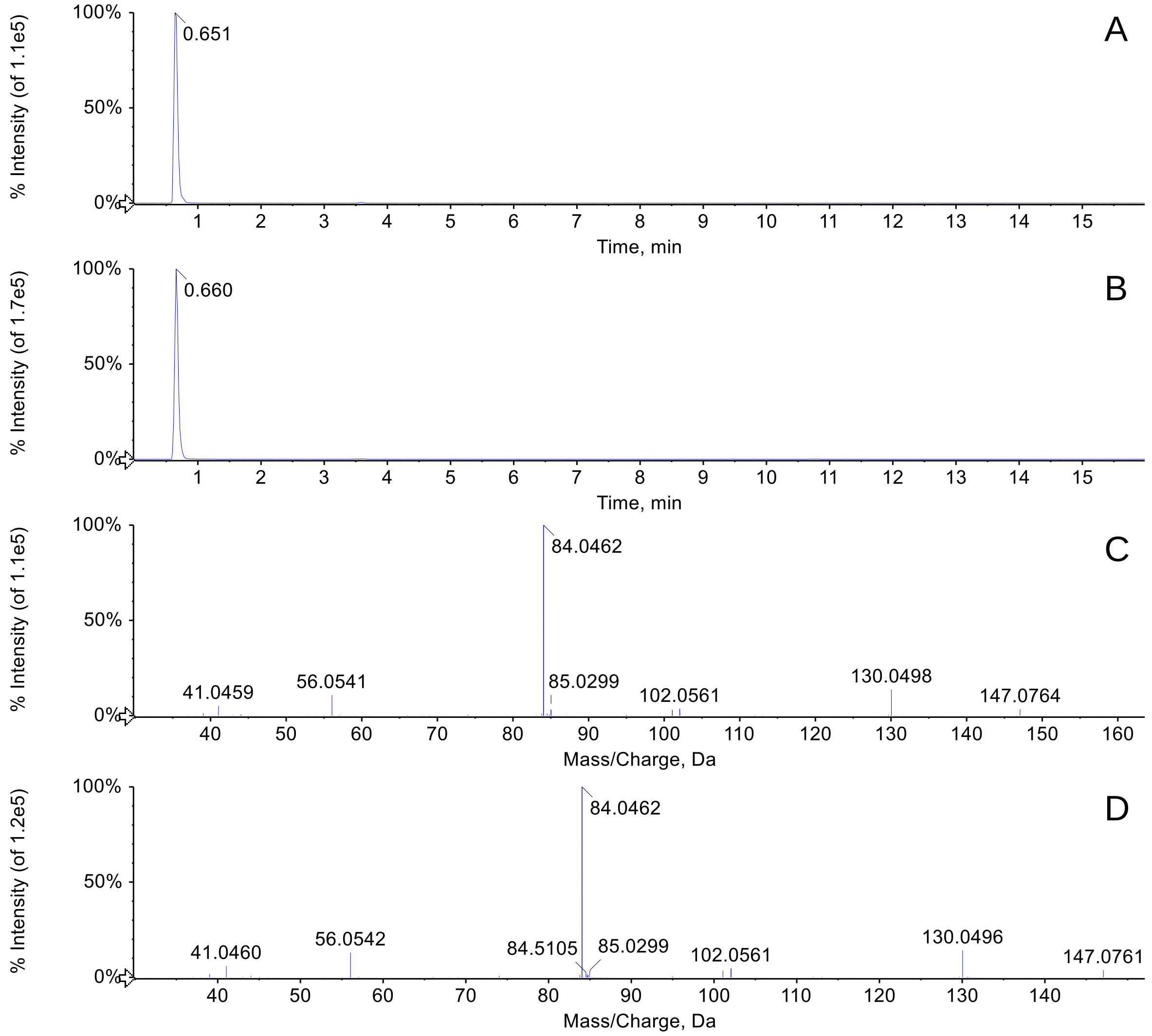

Fig S5: The EIC and MS/MS spectra for **glutamine** from the reference substance (A and C) and serum sample (B and D)

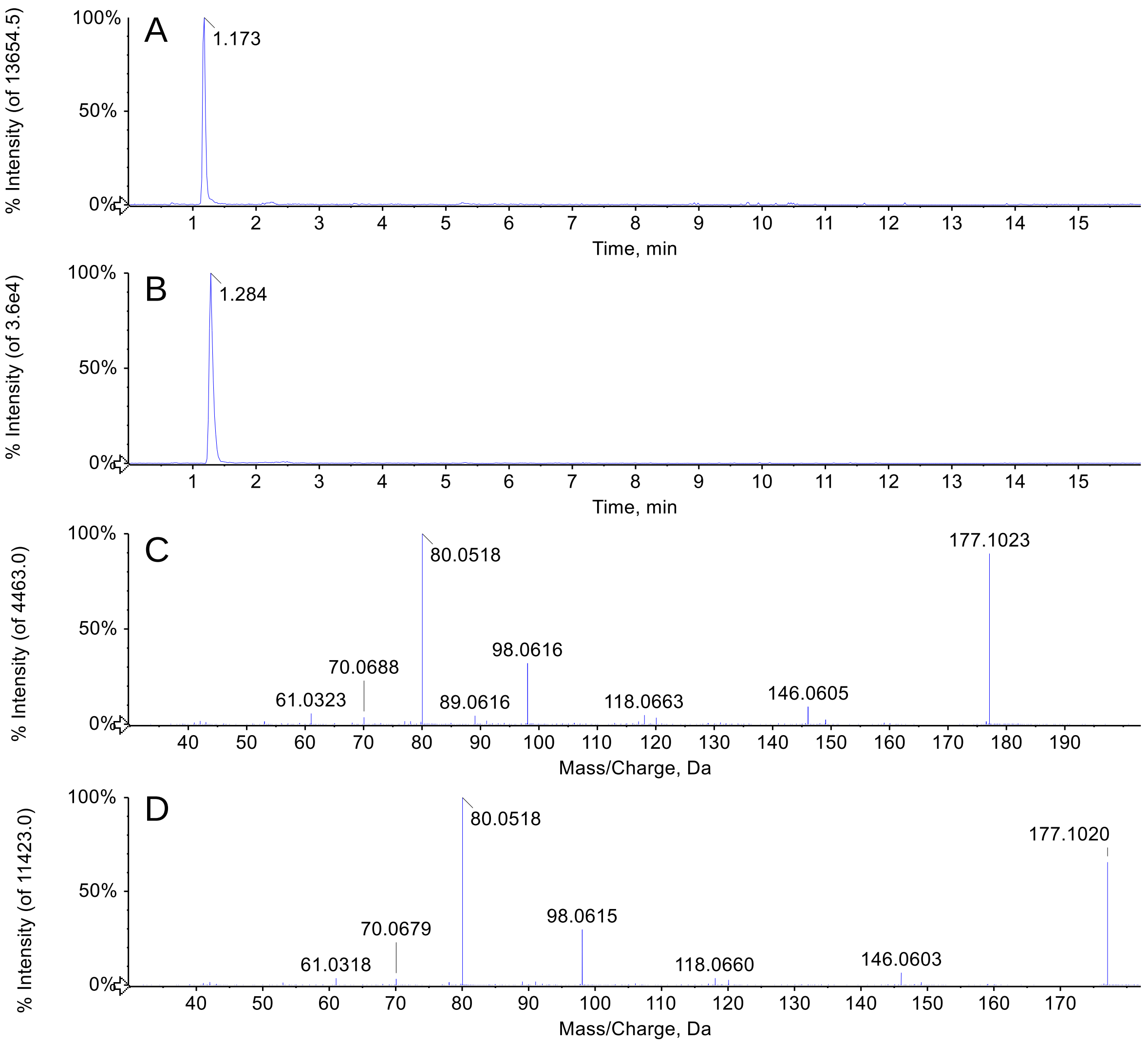

Fig S6: The EIC and MS/MS spectra for **5-hydroxytryptamine** from the reference substance (A and C) and serum sample (B and D)

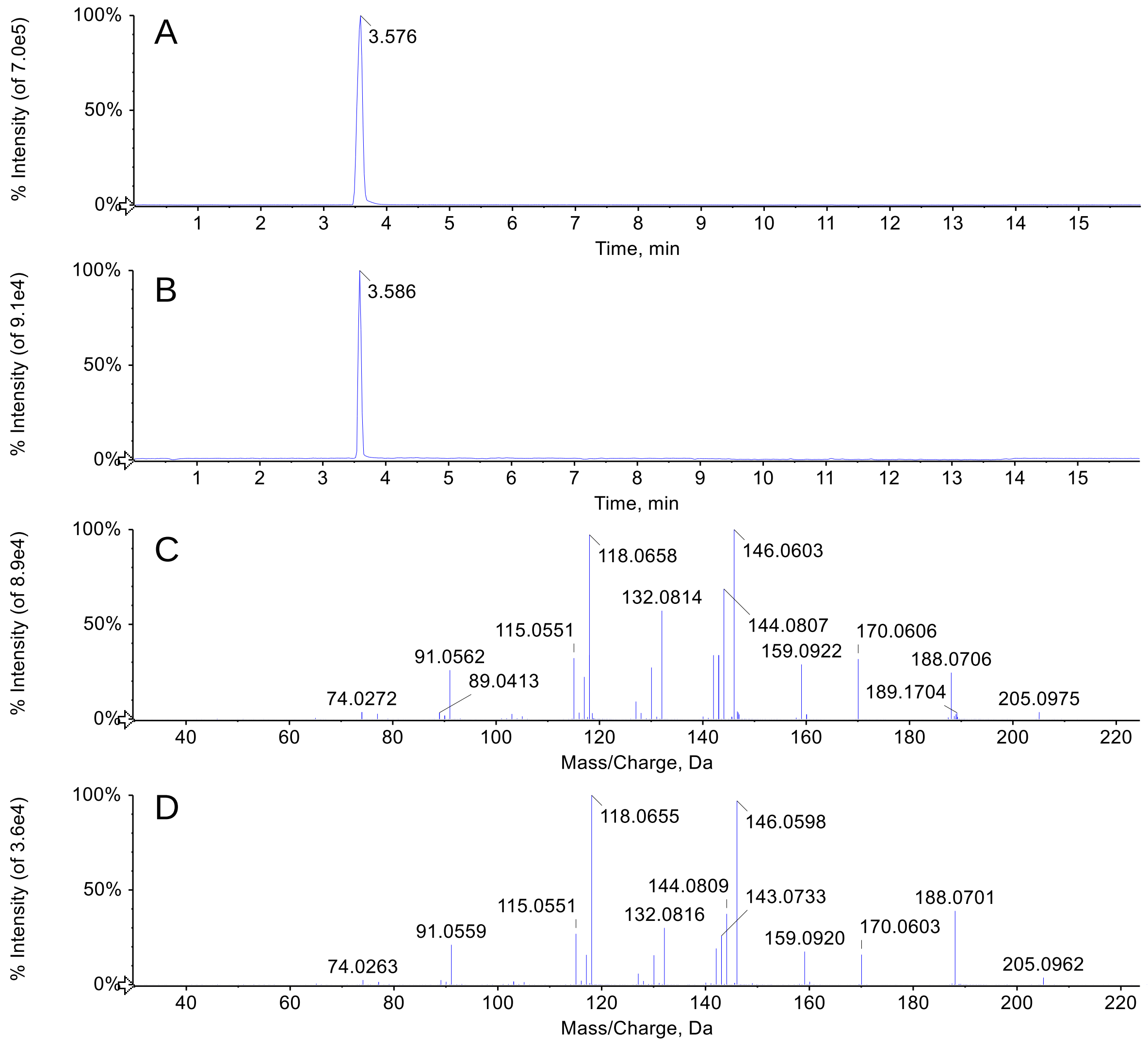

Fig S7: The EIC and MS/MS spectra for **tryptophan** from the reference substance (A and C) and serum sample (B and D)

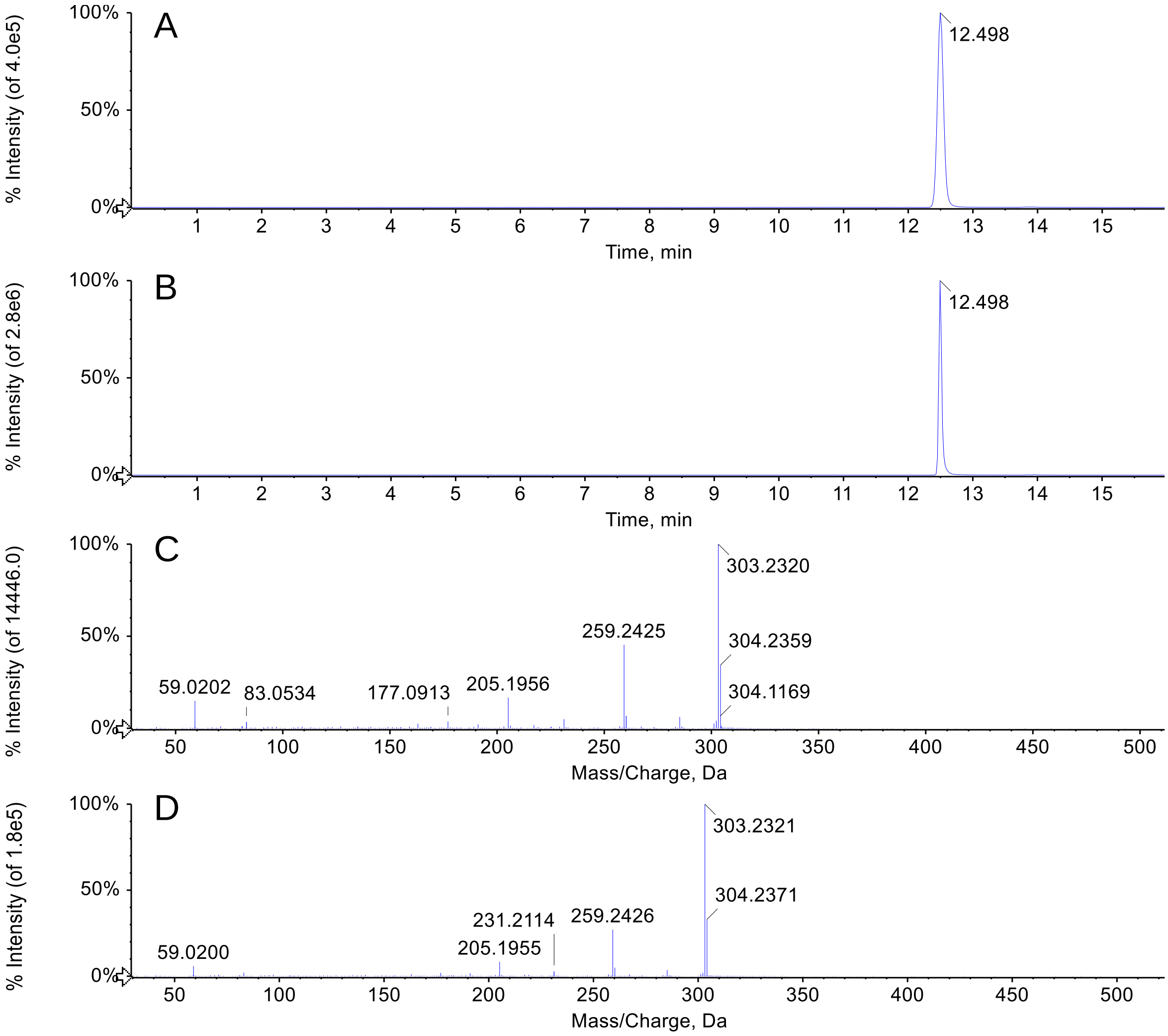

Fig S8: The EIC and MS/MS spectra for **arachidonic acid** from the reference substance (A and C) and serum sample (B and D)

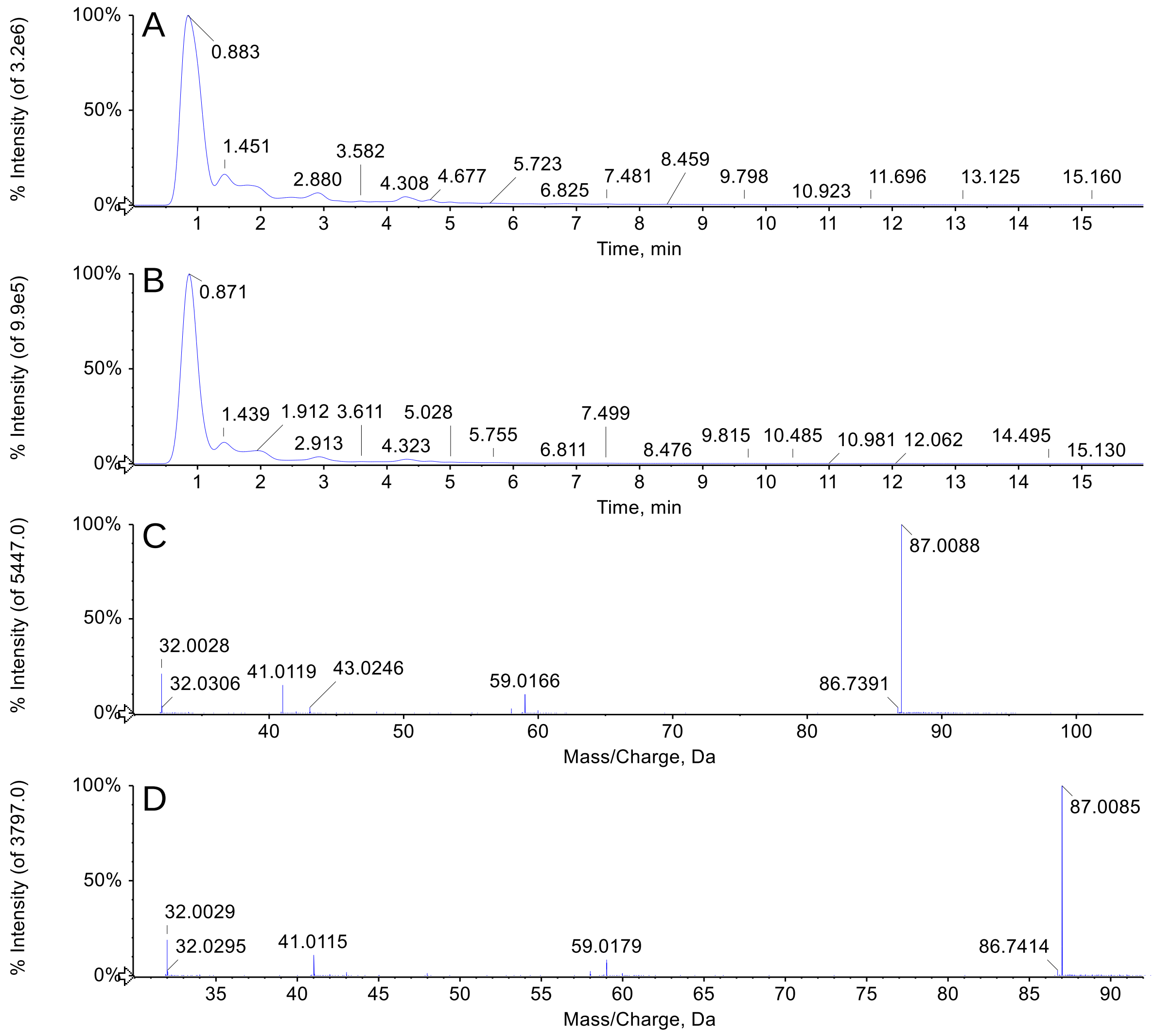

Fig S9: The EIC and MS/MS spectra for **pyruvic acid** from the reference substance (A and C) and serum sample (B and D)

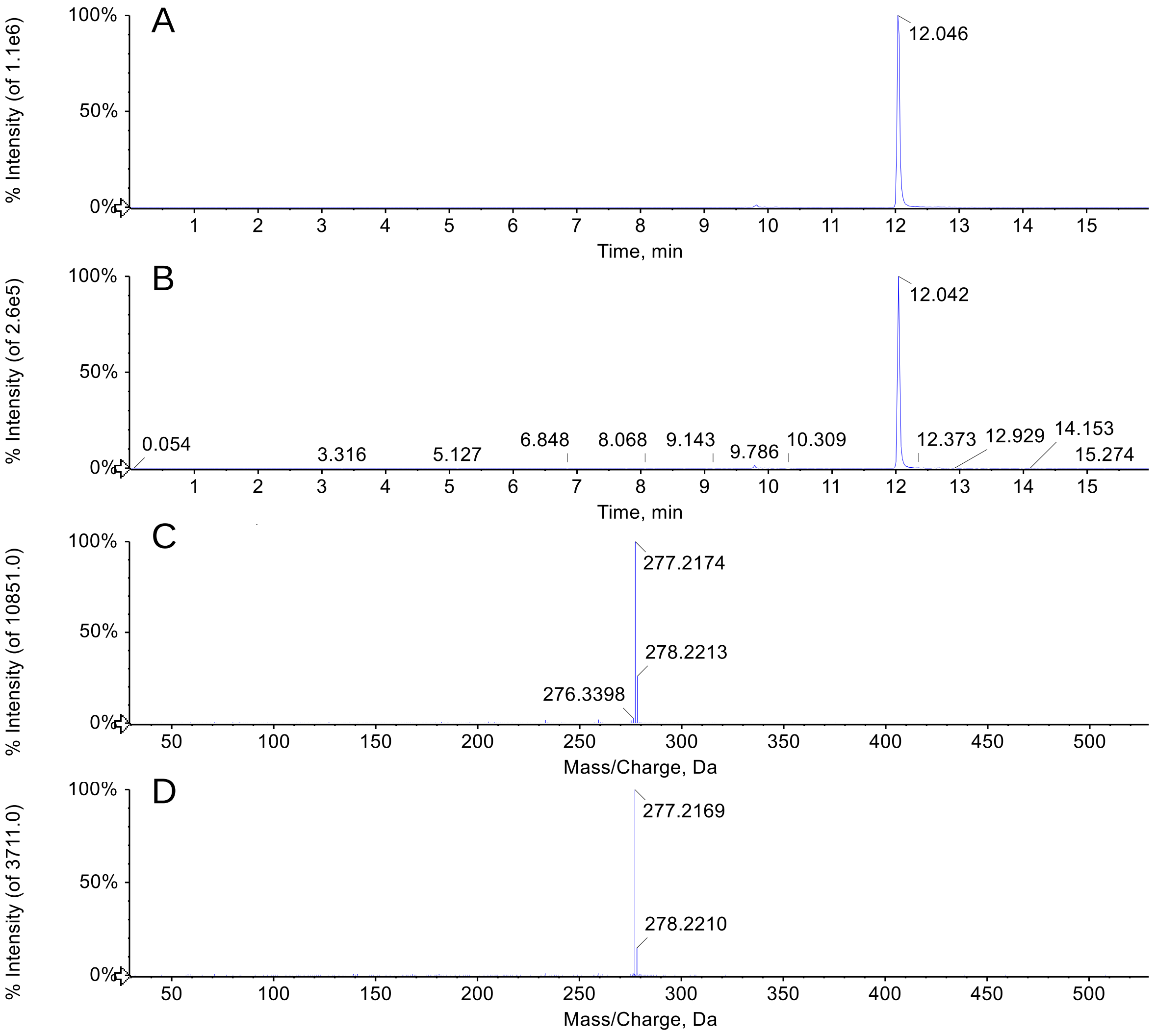

Fig S10: The EIC and MS/MS spectra for **α-linolenic acid** from the reference substance (A and C) and serum sample (B and D)

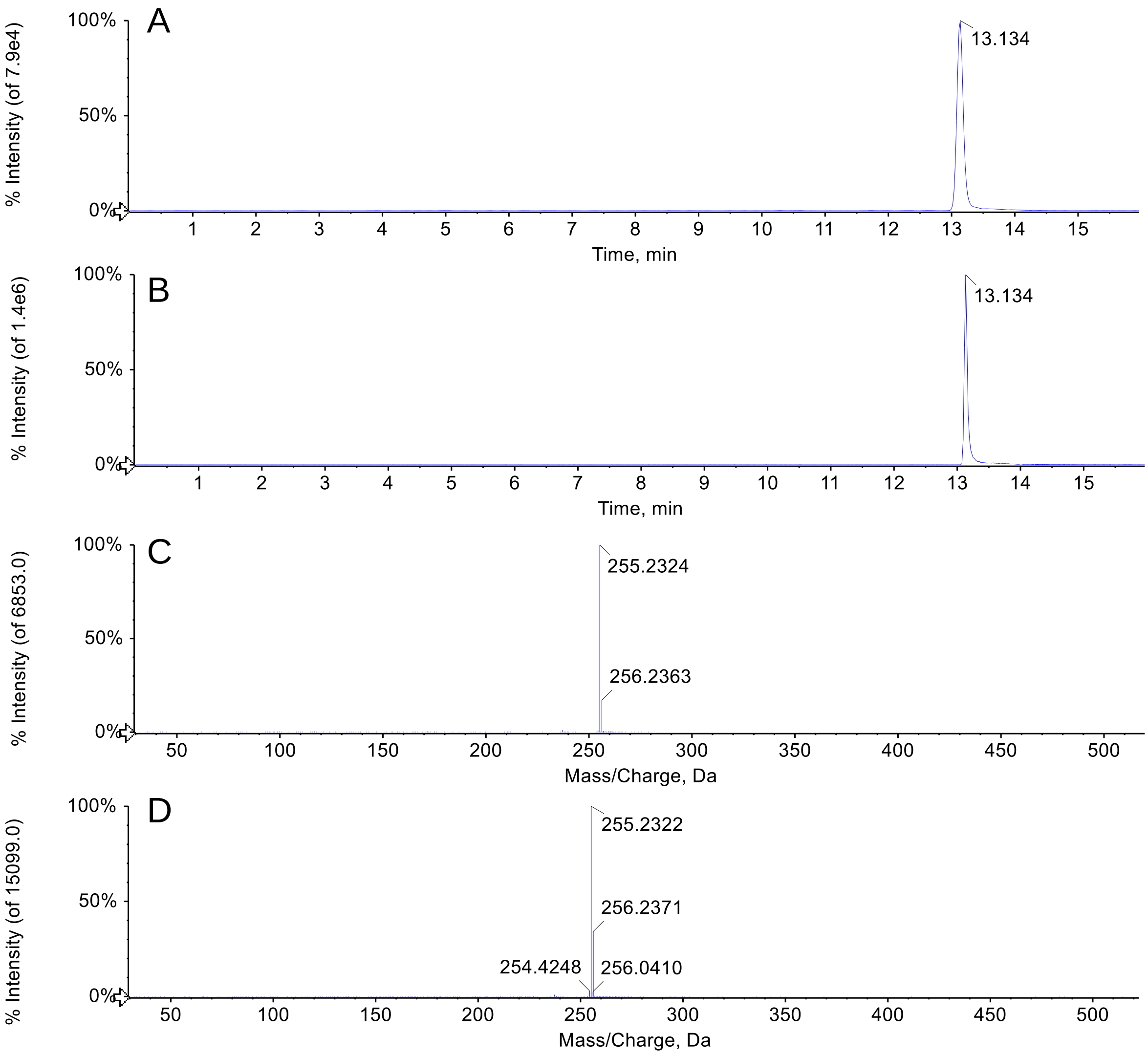

Fig S11: The EIC and MS/MS spectra for **palmitic acid** from the reference substance (A and C) and serum sample (B and D)

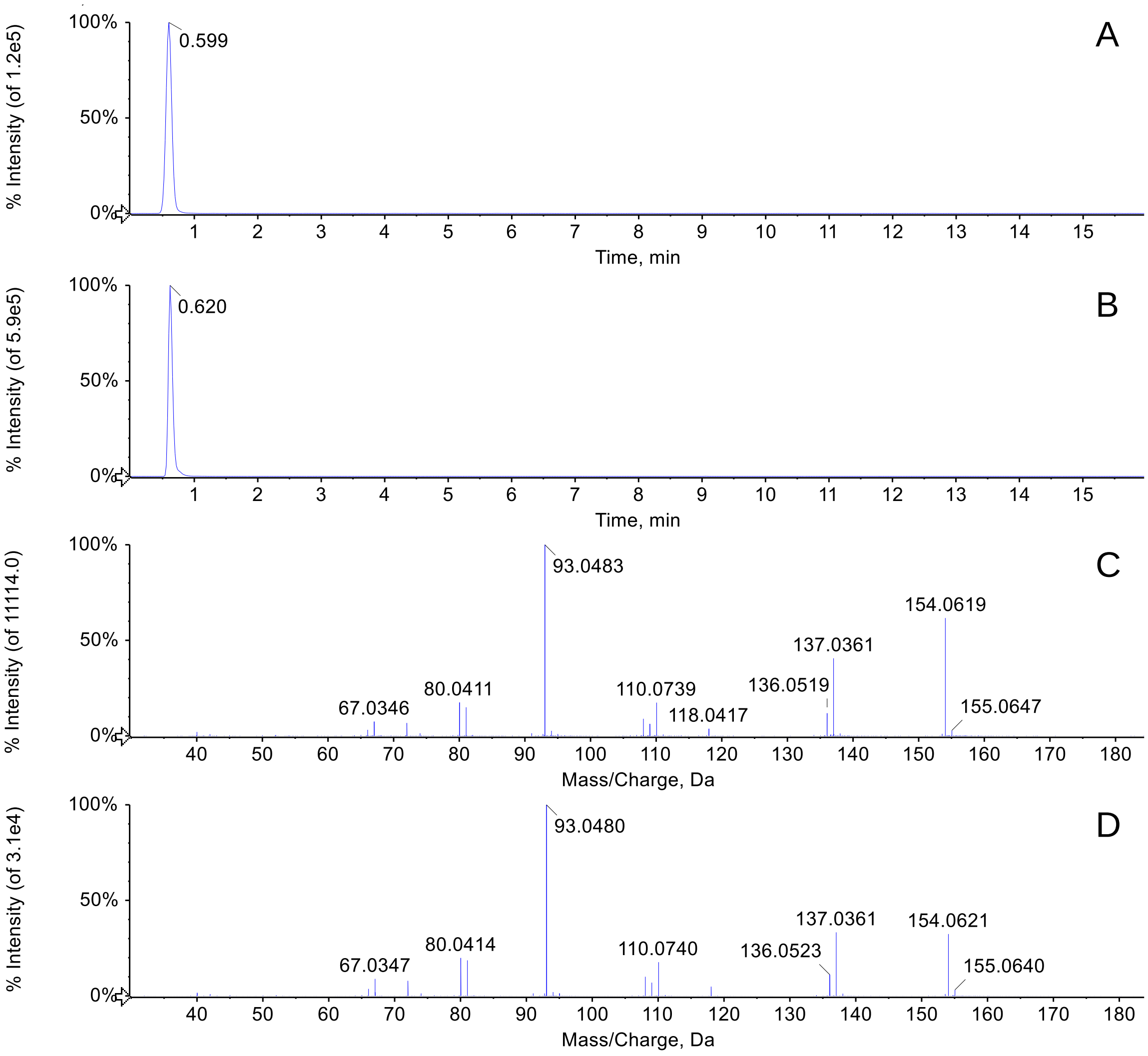

Fig S12: The EIC and MS/MS spectra for **histidine** from the reference substance (A and C) and serum sample (B and D)
